# Protonation‐Driven Polarization Retention Failure in Nano‐Columnar Lead‐Free Ferroelectric Thin Films

**DOI:** 10.1002/advs.202408784

**Published:** 2024-11-03

**Authors:** Muhammad Sheeraz, Chang Won Ahn, Nguyen Xuan Duong, Soo‐Yoon Hwang, Ji‐Soo Jang, Eun‐Young Kim, Yoon Ki Kim, Jaeyeong Lee, Jong Sung Jin, Jong‐Seong Bae, Myang Hwan Lee, Hyoung‐Su Han, Gi‐Yeop Kim, Shinuk Cho, Tae Kwon Song, Sang Mo Yang, Sang Don Bu, Seung‐Hyub Baek, Si‐Young Choi, Ill Won Kim, Tae Heon Kim

**Affiliations:** ^1^ Department of Physics and Energy Harvest‐Storage Research Center (EHSRC) University of Ulsan Ulsan 44610 Republic of Korea; ^2^ Department of Materials Science and Engineering Pohang University of Science and Technology Pohang 37673 Republic of Korea; ^3^ Electronic Materials Research Center Korea Institute of Science and Technology Seoul 02792 Republic of Korea; ^4^ Department of Physics Research Institute of Physics and Chemistry Jeonbuk National University Jeonju 54896 Republic of Korea; ^5^ Department of Physics Sogang University Seoul 04107 Republic of Korea; ^6^ Busan Center Korea Basic Science Institute (KBSI) Busan 46742 Republic of Korea; ^7^ School of Materials Science and Engineering Changwon National University Gyeongnam 51140 Republic of Korea; ^8^ School of Materials Science and Engineering University of Ulsan Ulsan 44776 Republic of Korea; ^9^ Division of Nano & Information Technology KIST School University of Science and Technology Seoul 02792 Republic of Korea; ^10^ Center for Van der Waals Quantum Solids Institute for Basic Science Pohang 37673 Republic of Korea; ^11^ Department of Semiconductor Engineering Pohang University of Science and Technology Pohang 37673 Republic of Korea

**Keywords:** ferroelectric, (K,Na)NbO_3_, thin film, polarization retention loss, epitaxy

## Abstract

Understanding microscopic mechanisms of polarization retention characteristics in ferroelectric thin films is of great significance for exploring unusual physical phenomena inaccessible in the bulk counterparts and for realizing thin‐film‐based functional electronic devices. Perovskite (K,Na)NbO_3_ is an excellent class of lead‐free ferroelectric oxides attracting tremendous interest thanks to its potential applications to nonvolatile memory and eco‐friendly energy harvester/storage. Nonetheless, in‐depth investigation of ferroelectric properties of (K,Na)NbO_3_ films and the following developments of nano‐devices are limited due to challenging thin‐film fabrication associated with nonstoichiometry by volatile K and Na atoms. Herein, ferroelectric (K,Na)NbO_3_ films of which the atomic‐level geometrical structures strongly depend on thickness‐dependent strain relaxation are epitaxially grown. Nanopillar crystal structures are identified in fully relaxed (K,Na)NbO_3_ films to the bulk states representing a continuous reduction of switchable polarization under air environments, that is, polarization retention failures. Protonation by water dissociation is responsible for the humidity‐induced retention loss in nano‐columnar (K,Na)NbO_3_ films. The protonation‐driven polarization retention failure originates from domain wall pinning by the accumulation of mobile hydrogen ions at charged domain walls for effective screening of polarization‐bound charges. Conceptually, the results will be utilized for rational design to advanced energy materials such as photo‐catalysts enabling ferroelectric tuning of water splitting.

## Graphical abstract

This work presents a protonation effect on polarization retention properties in lead‐free ferroelectric (K,Na)NbO_3_ thin films exhibiting nanopillar geometry. The polarization retention loss in nano‐columnar (K,Na)NbO_3_ thin films is attributed to domain wall pinning by the accumulation of hydrogen ions arising from water dissociations in air, returning to the initial state through thermally activated deprotonation.

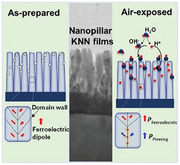

## 1 Introduction

Ferroelectric (FE) materials, where two spontaneous polarizations are energetically equivalent and these non‐zero polarization states are switchable by the application of an external electric field,^[^ ^1^ ^]^ are of practical interest due to their potential applications to multi‐functional devices such as non‐volatile FE random access memory,^[^ ^2^, ^3^ ^]^ FE field‐effect transistors,^[^ ^3^, ^4^ ^]^ topologically protected quantum states (e.g., ferroelectric vortices^[^ ^5^, ^6^ ^]^ and polar Skyrmions^[^ ^7^ ^]^), electromechanical sensors/actuators/nanogenerators,^[^ ^8^, ^9^, ^10^ ^]^ and renewable energy harvesters/storages.^[^ ^11^, ^12^, ^13^ ^]^ In particular, polarization retention properties (i.e., the ability of FE materials to maintain their polarization in the absence of external electric bias)^[^ ^14^, ^15^ ^]^ have been extensively examined to evaluate the operational performance and reliability of FE thin‐film‐based functional devices.^[^ ^15^ ^]^ For the last decades, numerous efforts have been made to understand the polarization retention characteristics and to elucidate the microscopic origin in lead‐based FE thin films (e.g., Pb(Zr,Ti)O_3_).^[^ ^15^, ^16^, ^17^, ^18^ ^]^ However, a detailed study on the polarization retention loss behaviors of lead‐free FE thin films has been rare due to the challenging synthesis of epitaxial lead‐free thin films exhibiting monolithic structures.^[^ ^19^, ^20^, ^21^ ^]^

Perovskite (K,Na)NbO_3_ (KNN) is a promising candidate for lead‐free FE oxide materials as an alternative to lead‐based FEs owing to its excellent physical properties such as the high Curie temperature (*T*_C_ ≈ 420 °C) and moderate polarization (18–33 µC cm^−2^) comparable to that (20–47 µC cm^−2^) of Pb(Zr,Ti)O_3_.^[^ ^12^, ^22^, ^23^ ^]^ KNN is bio‐compatible with no harmful element to human bodies. Namely, it is free from global regulations of toxic elements (e.g., Pb) such as the Restriction of Hazardous Substances Directive (RoHS), which allows us to achieve environment‐friendly and sustainable developments of functional electronic devices.^[^ ^20^ ^]^ Despite the fascinating physical properties, systematic studies on polarization retention nature in lead‐free KNN thin films have been limited, because the retention capability is directly degenerated by leakage currents arising from vacancy defects formed by the volatilization of alkali K and Na elements.^[^ ^20^, ^24^, ^25^ ^]^ In the presence of K and Na vacancies with an effective negative charge, positive oxygen vacancies are also created for charge neutrality.^[^ ^26^ ^]^ Note that these non‐neutral ionic vacancies are usually mobile and thereby, the movement of the charged defects can substantially modify typical FE properties (i.e., remnant polarization and coercive field) leading to both suppression of FE polarization by charge screening^[^ ^27^, ^28^ ^]^ and/or domain wall pinning,^[^ ^29^, ^30^, ^31^ ^]^ and electrical leakage in transport characteristics.^[^ ^19^, ^26^ ^]^ Although the polarization retention behaviors in lead‐free FE KNN are closely linked to the formation and migration of free charge carriers attributed to off‐stoichiometry,^[^ ^14^ ^]^ the underlying mechanism of the polarization retention loss is still unclear and rather under debate. Therefore, it is highly essential to clearly show how the non‐neutral charges produce the retention failure of lead‐free FE KNN thin films on a microscopic scale.^[^ ^14^, ^18^, ^32^ ^]^

Protonation driven by hydrogen ions originating from water splitting can induce polarization retention failure of lead‐free KNN thin films. In functional oxide materials, a diverse range of physical properties are quite susceptible/vulnerable to humidity.^[^ ^28^, ^33^, ^34^, ^35^, ^36^ ^]^ For example, it is intriguing that low‐frequency dielectric constants in oxide compounds are remarkably enhanced by introducing hydrogen ions separated from water molecules into dielectric ceramics in a humidity condition.^[^ ^34^ ^]^ In oxygen‐deficient dielectric materials, it is energetically favorable that water molecules are adsorbed at given oxygen vacancies resulting in dissociation to hydrogen ions (H^+^) and hydroxyl ions (OH^−^).^[^ ^28^, ^37^ ^]^ Likewise, it is highly plausible that protons are generated in FE KNN films incorporating vacancy defects through water dissociation. When oxygen‐deficient KNN films are exposed to ambient air, chemisorbed water molecules at surface oxygen vacancy sites can be split to H^+^ and OH^−^ ions. Considering that the positive hydrogen ion (i.e., proton) is the smallest and lightest ion with high mobility,^[^ ^38^ ^]^ the separated protons from water molecules should be diffused into the KNN films both screening polarization bound charges^[^ ^28^ ^]^ and increasing electric leakage currents^[^ ^39^ ^]^ in conjunction with resultant polarization retention loss (Figure S1, Supporting Information).

In this work, we demonstrate a protonation effect on polarization retention behaviors in lead‐free FE KNN thin films with nanoscale thickness. To assess this, we fabricated epitaxial KNN/La_0.7_Sr_0.3_MnO_3_ (LSMO)/SrTiO_3_ (001) thin‐film heterostructures with two different KNN thicknesses. While thinner KNN (≈35 nm) films exhibited 2‐D planar structures analogous to conventional heteroepitaxial thin films, pillar structures were found in thicker KNN (≈600 nm) films at the atomic level. Intriguingly, for the 35‐nm‐thick KNN films with the layer structures, FE polarization was continuously retained with little variation, whereas a time‐dependent reduction of switchable polarization (i.e., polarization retention loss) was evident in the 600‐nm‐thick KNN films with cylindrical geometry. Various experimental analyses revealed that the observed retention loss in nano‐columnar KNN thin films was largely attributed to the effective screening of polarization bound charges and/or pinning of charged domain walls by the accumulation of mobile hydrogen ions dissociated from water molecules in the air. Furthermore, the suppressed hysteresis loop with the decreasing remnant polarization induced by proton diffusion was restored by deprotonation through a heat treatment. It was also identified that the suppression of FE hysteresis by protonation and the subsequent recovery by thermally activated deprotonation were reversible. Microscopic mechanisms of the protonation‐driven polarization retention failure were discussed along with potential developments of functional energy materials in which a hydrogen evolution reaction by water splitting would be tunable via polarization reversal.

## 2 Results and Discussion

**Figure 1:**
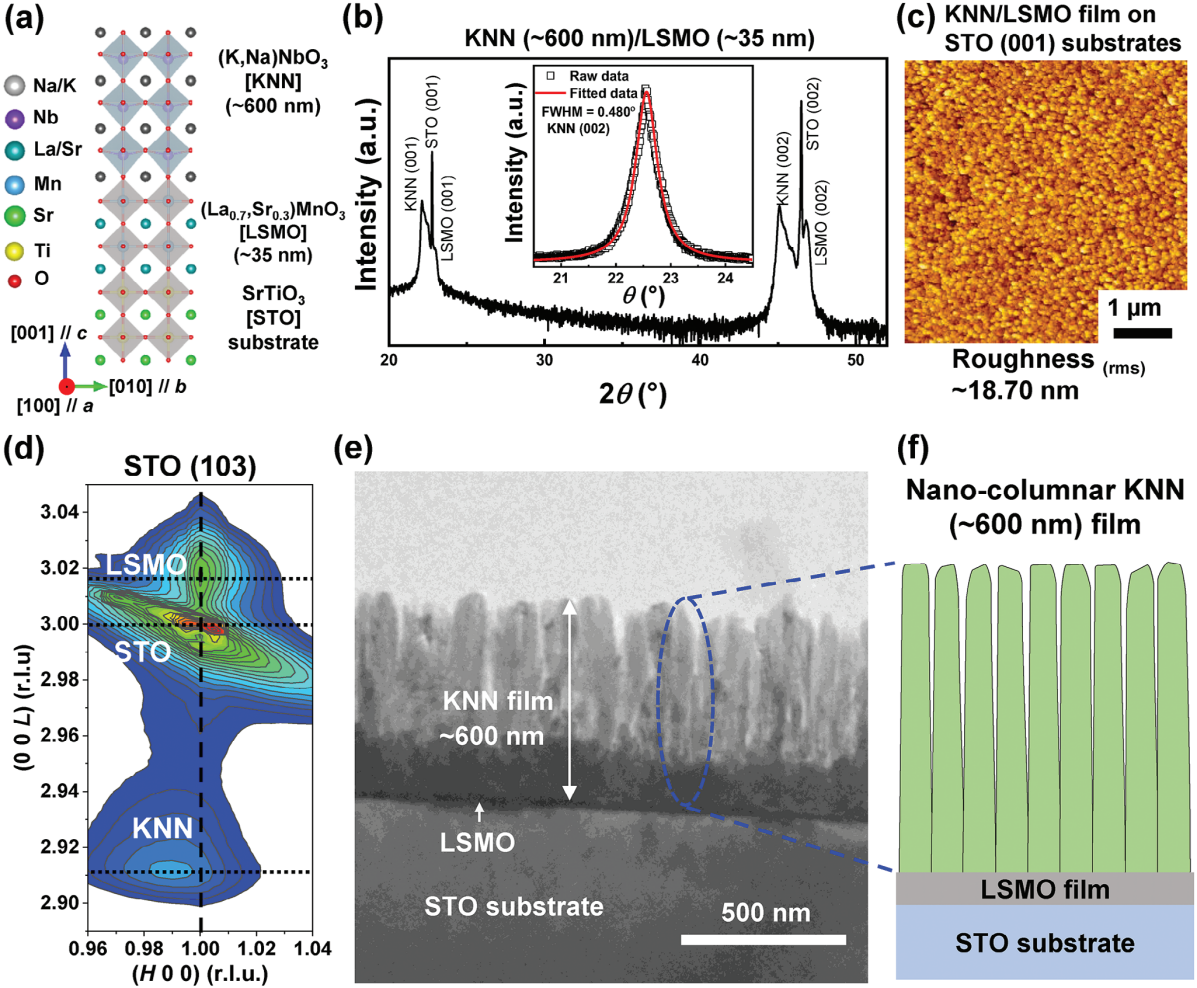
Epitaxial KNN/LSMO thin film heterostructures grown on SrTiO_3_ (001) substrates. a) Schematic 2D diagram of the 600 nm‐thick KNN/LSMO heterostructure films on SrTiO_3_ (001) substrates. Note that the bulk pseudocubic (pc) lattice constants of the KNN single crystal are *a*_pc_ = 3.976, *b*_pc_ = 3.932, and *c*_pc_ = 3.969 Å.^[^ ^40^ ^]^ b) The XRD analyses of the as‐grown KNN (≈600 nm)/LSMO (≈35 nm) hetero‐bilayer thin films. From the rocking‐curve measurement of the thicker KNN (002) Bragg peak in the inset of Figure 1b, we identified that the FWHM was ≈0.48°, nearly twice (≈0.30°) to 600 nm‐thick KNN/LSMO (≈15 nm) thin films epitaxially grown on SrTiO_3_ (001) substrates (inset in the Figure S2, Supporting Information). c) The AFM topography image of the KNN (≈600 nm)/LSMO (≈35 nm) films on SrTiO_3_ substrates. d) High‐resolution RSMs of pure KNN (≈600 nm)/LSMO (≈35 nm) films around the (103) Bragg peaks of SrTiO_3_ (001) substrates. e) The cross‐sectional STEM image of the as‐grown KNN (≈600 nm)/LSMO (≈35 nm) films on the SrTiO_3_ substrates where the f) nano‐columnar KNN film is shown in the enlarged view. The scale bar in the STEM image is 500 nm.

To evaluate the structural properties of the as‐grown lead‐free KNN thin films, we carried out the x‐ray diffraction (XRD) analyses of KNN/LSMO bilayer films with two different KNN thickness (i.e., 35 and 600 nm) epitaxially grown on SrTiO_3_ (001) substrates, as shown in Figure S2a, Supporting Information and **Figure** **1**a, respectively. Based on the XRD *θ*−2*θ* results (Figure 1b), we confirmed that the thicker KNN (≈600 nm)/LSMO (≈35 nm) bilayer films were epitaxial with no secondary phase (XRD patterns of the thinner KNN (≈35 nm)/LSMO (≈15 nm) films on STO (001) substrates shown in Figure S2a, Supporting Information). The measured full width at half maximum (FWHM) values of the thicker KNN (≈600 nm) films were ≈0.48° (the rocking curve data in the inset of Figure 1b larger than those (≈0.30°) of the thinner KNN films (the rocking curve data in the inset of Figure S2a, Supporting Information). Atomic force microscopy (AFM) images of the as‐grown KNN films showed that the surface roughness (≈18.7 nm) of the 600‐nm‐thick KNN films was larger than that (≈16.1 nm) of the 35 nm‐thick KNN films probably due to thickness‐dependent strain relaxation (Figure 1c and Figure S2b, Supporting Information).

The thickness‐dependent strain relaxation in the KNN/LSMO films was also verified by the high‐resolution reciprocal space mappings (RSMs) around the (103) Bragg peaks of cubic SrTiO_3_ substrates, as shown in Figure 1d. It was evident that the positions of KNN diffraction peaks deviated from the vertical line (denoted by the black dashed line) while the LSMO peaks were in a line with the same *H* values as the SrTiO_3_ substrates. The in‐plane (*a*) and out‐of‐plane (*c*) lattice parameters of the KNN (LSMO) layers were estimated to *a*_KNN_ = 3.945 (*a*_LSMO_ = 3.905) and *c*_KNN_ = 4.001 Å (*c*_LSMO_ = 3.871 Å) for the thicker (≈600 nm) KNN films, respectively. Meanwhile, in the thinner (≈35 nm) KNN films, all *H* values of KNN (*a*_KNN_ = 3.905 and *c*_KNN_ = 3.993 Å), LSMO (*a*_LSMO_ = 3.905 and *c*_LSMO_ = 3.899 Å), SrTiO_3_ (*a*_SrTiO3_ = 3.905 and *c*_SrTiO3_ = 3.905 Å) diffraction peaks were identical with little variation indicative of the in‐plane lattice coherency (Figure S2c and Table S1, Supporting Information). Namely, the 35‐nm‐thick KNN films were completely under in‐plane compressive strain with respect to the underlying SrTiO_3_ substrates with volume contraction by cation vacancy formation.^[^ ^40^ ^]^ With the increasing film thickness, the initial in‐plane compressive strain would be relieved and thereby, the resulting in‐plane and out‐of‐plane lattice constants should get closer to bulk values.^[^ ^41^ ^]^ Considering that the pseudocubic in‐plane and out‐of‐plane lattice constants of bulk KNN were *a*_pc_ = 3.932 and *c*_pc_ = 3.969 Å, respectively (Table S1, Supporting Information),^[^ ^42^ ^]^ the thicker (≈600 nm) KNN films were nearly relaxed to the bulk state. In addition, we note that the calculated unit cell volumes (≈62.27 Å^3^) of the thicker (≈600 nm) KNN films were slightly larger than the bulk volume (≈62.06 Å^3^) implying oxygen‐vacancy‐induced volume expansion in epitaxial oxide thin films.^[^ ^40^, ^42^ ^]^

The geometrical lattice structures on a nanoscale strongly depended on the strain states of the as‐grown lead‐free FE thin films. To microscopically visualize the atomic‐level lattice structures of our KNN films with two different strain states, we performed annular bright field (ABF) scanning transmission electron microscopy (STEM) measurements of 35‐ and 600‐nm‐thick KNN films, as shown in Figure S2d, Supporting Information and Figure 1e, respectively. The obtained ABF images along the [100] zone‐axis revealed that atomic‐scale lattice structures were quite different with the increasing thickness of the KNN film layers. While the thinner (≈35 nm) KNN films under in‐plane compressive strain displayed 2‐D planar geometry similar to typical oxide thin films, unusual nano‐columnar structures emerged in the thicker (≈600 nm) KNN films with fully relaxed strain states. The magnified cross‐sectional STEM images evidenced that the width of the observed nanopillars was ≈3 nm (Figure S3, Supporting Information), which was reminiscent of a previous report of nanopillar structures in Na‐deficient NaNbO_3_ films.^[^ ^43^ ^]^ Note that the 600‐nm‐thick KNN films exhibiting the bulk strain state incorporated a certain amount of oxygen vacancies with volume expansion and hence, K and Na vacancies would be also formed inside the films for charge neutrality.^[^ ^26^ ^]^ Considering that the nanoscale lattice structures of complex oxide thin films were very susceptible to the strain‐mediated non‐stoichiometry,^[^ ^44^ ^]^ the observed nano‐columnar structures of our KNN films should be involved with off‐stoichiometry of volatile K/Na cations and the successive formation of vacancy defects.

**Figure 2:**
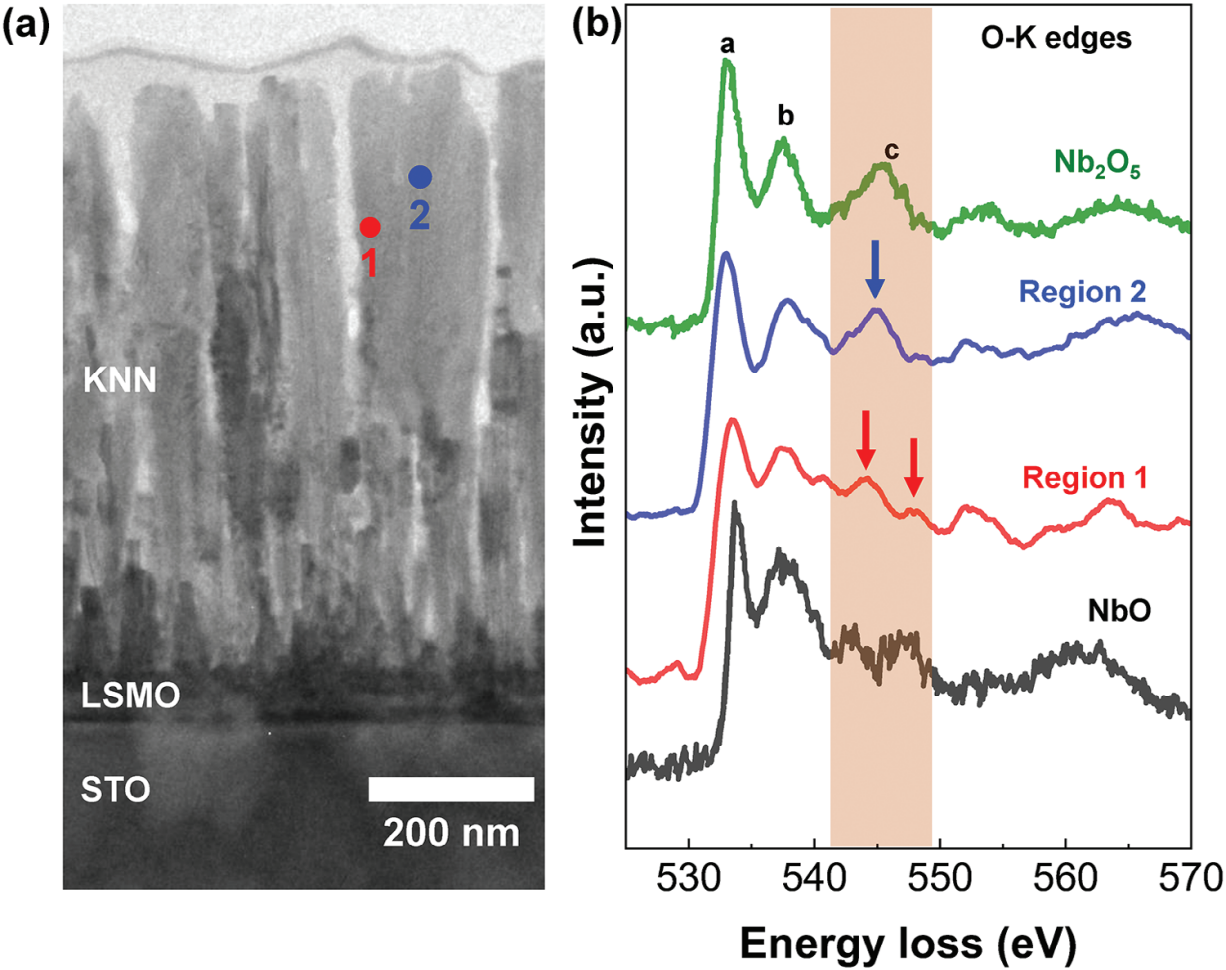
a) The cross‐sectional STEM image of the as‐grown KNN (≈600 nm)/LSMO (≈35 nm) films on the STO substrates with a clear nanopillar geometry. The scale bar in the STEM image is 200 nm. b) The O‐*K* edge of the EELS spectra (i.e., the peak “c”) between regions 1 and 2 of the nano‐columnar KNN films. In the shaded rectangular region marked by the peak “c” near the electron energy loss of 545 eV, the 2 peaks highlighted by 2 red arrows in the red spectrum are associated with region 1 whereas a single peak highlighted by a blue arrow was linked to spectra of region 2. The reference spectra of NbO and Nb_2_O_5_ spectra are shown in black and green colors, respectively.^[^ ^45^ ^]^

To experimentally verify the presence of oxygen vacancies in the nanopillar KNN films, we further extended our STEM analyses of 600‐nm‐thick KNN films by performing electron energy loss spectroscopy (EELS) at O‐*K* edges. In the 600‐nm‐thick KNN films with nano‐columnar structures, we selected two different regions for the EELS measurements, as shown in **Figure** **2**a. Specifically, the first spot was in the proximity of the edges of the nano‐columnar structures (i.e., outer surfaces of the nanopillars, Region 1 marked by a red solid circle) and the second spot was positioned inside the nanopillars (Region 2 marked by a blue solid circle). In general, the O‐*K* edge EELS spectra in perovskite oxides mainly consisted of 3 peaks, as indicated by “a”, “b”, and “c” in Figure 2b. Herein, the “a” and “c” peaks were closely related to B‐site atoms in the ABO_3_ perovskite oxides, while the “b” peaks were solely dependent on A‐site atoms.^[^ ^45^ ^]^ Especially, the “c” peak nature was governed by the characteristics of hybridized orbitals between B and O ions, and thereby, oxygen vacancies in our KNN films were more likely to modify the spectral feature of the “c” peak susceptible to a change of the Nb‐O hybridization.

It was highly likely that more oxygen vacancies were accumulated at the outer surfaces of nano‐columnar structures in 600‐nm‐thick KNN films than in the inner regions. At the edges of nano‐columnar structures (Region 1), the thicker KNN films exhibited double peak behaviors in the obtained O‐*K* edge EELS spectra (denoted by 2 red arrows in Figure 2b) in the energy range of 542 to 548 eV. On the contrary, inside the nano‐columnar structures (Region 2), a single peak (denoted by a blue arrow in Figure 2b) was observed in the O‐*K* edge EELS spectra for the same energy range. As aforementioned, such a discrepancy in the spectral feature of the peak “c” would be attributed to different bonding (i.e., hybridization) characteristics between the transition metal Nb ions and oxygen ions.^[^ ^45^ ^]^ In fact, the double peak characteristics with lower peak intensity observed in Region 1 were close to the spectral shapes (the black spectra in Figure 2b) of oxygen‐deficient NbO.^[^ ^45^ ^]^ Meanwhile, the observed single peak in Region 2 corresponded to the EELS peak (the green spectra in Figure 2b) of oxygen‐rich Nb_2_O_5_.^[^ ^45^ ^]^ It was therefore plausible that more concentration of oxygen vacancies was accumulated at the nanopillar boundaries (Region 1, the characteristic bonding type analogous to oxygen‐deficient NbO) due to a large variety of disorders compared with the nanopillar grains (Region 2, the characteristic bonding type comparable to oxygen‐abundant Nb_2_O_5_) of the 600‐nm‐thick KNN films.^[^ ^43^, ^44^, ^45^ ^]^ Accordingly, the oxygen vacancy distribution in the thicker KNN films with nano‐columnar structures should be more inhomogeneous spatially than the thinner KNN films with 2D planar geometry. Considering that the oxygen vacancies were mostly located at the nanopillar boundaries rather than inside the nanopillar grains, it followed that the thicker KNN films with the nano‐columnar structures incorporated more amount of oxygen vacancies compared to the thinner KNN films with the planar structures.

**Figure 3:**
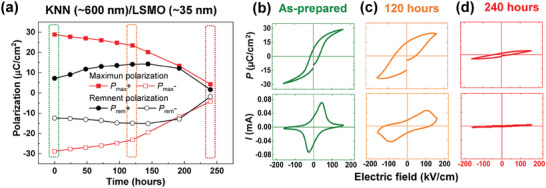
Extracted maximum (*P*_max_+ and *P*_max_−) and remnant (*P*_rem_+ and *P*_rem_−) polarization values of the epitaxial KNN (≈600 nm)/LSMO (≈35 nm) hetero‐bilayer thin films on SrTiO_3_ substrates with varying time (i.e., the polarization values of the KNN films were obtained up to 240 h). The polarization values of the as‐prepared, air exposure for 120 h and air exposure of 240 h were highlighted with green, orange, and red rectangles, respectively in Figure 3a. The polarization‐electric field (*P*‐*E*) hysteresis loops and the corresponding switching current (*I*‐*E*) loops of the KNN (≈600 nm)/LSMO (≈35 nm) hetero‐bilayer thin films in the b) as‐grown state (green color), c) at 120 h (orange color), and d) 240 h (red color).

To examine the effect of nanoscale crystal structures on FE properties in lead‐free KNN films, we monitored the time‐dependent evolution of FE hysteresis and switching current characteristics, as depicted in **Figure** **3**. To implement this, 35‐ and 600‐nm‐thick KNN films with different lattice structures were exposed to air at room temperature over time (up to 10 days; 240 h) and then, repeatedly measured the corresponding polarization (*P*)‐electric field (*E*) hysteresis loops and switching current (*I*‐*E*) curves (Figure S4, Supporting Information). It is interesting that the remnant and maximum polarization gradually decreased in the thicker KNN films with nanopillar structures as a function of time (Figure S5, Supporting Information). Such a reduction of FE polarization (i.e., polarization retention loss) was predominant in the extracted *P*‐*E* and *I*‐*E* behaviors of three different states, that is, the as‐prepared KNN films (highlighted by a green color), and the treated KNN films in air exposure for 120 (highlighted by an orange color) and 240 h (highlighted by a red color). Note that the measured remnant polarization in the air‐exposed KNN films for 120 h was slightly larger than the as‐prepared KNN films, which was not an intrinsic effect by enhancement of FE polarization. Rather, it was likely that the increasing leakage currents in the air‐exposed states extrinsically contributed to the increase of remnant polarization compared with the as‐prepared states.^[^ ^46^ ^]^ By contrast, no polarization retention loss was detected in the thinner KNN films with planar structures. Herein, remanent and maximum polarization were almost constant with little variation (Figures S6 and S7, Supporting Information). Moreover, we monitored the time‐dependent evolution of ferroelectric (FE) hysteresis for both the 35 nm and 600 nm‐thick KNN films without air exposure (i.e., Keeping the KNN films in a vacuum chamber) for every 24 h (Figure S8a, Supporting Information). The measured remnant and maximum polarization values remained nearly constant as a function of time, and no polarization retention loss was observed in both the 2D planar (≈35 nm) and the nanopillar (≈600 nm) films for 10 days (240 h) (Figure S8b,c, Supporting Information). This indicated that water molecules in the air were closely associated with the polarization retention failure in our KNN films with nano‐columnar structures.

**Figure 4:**
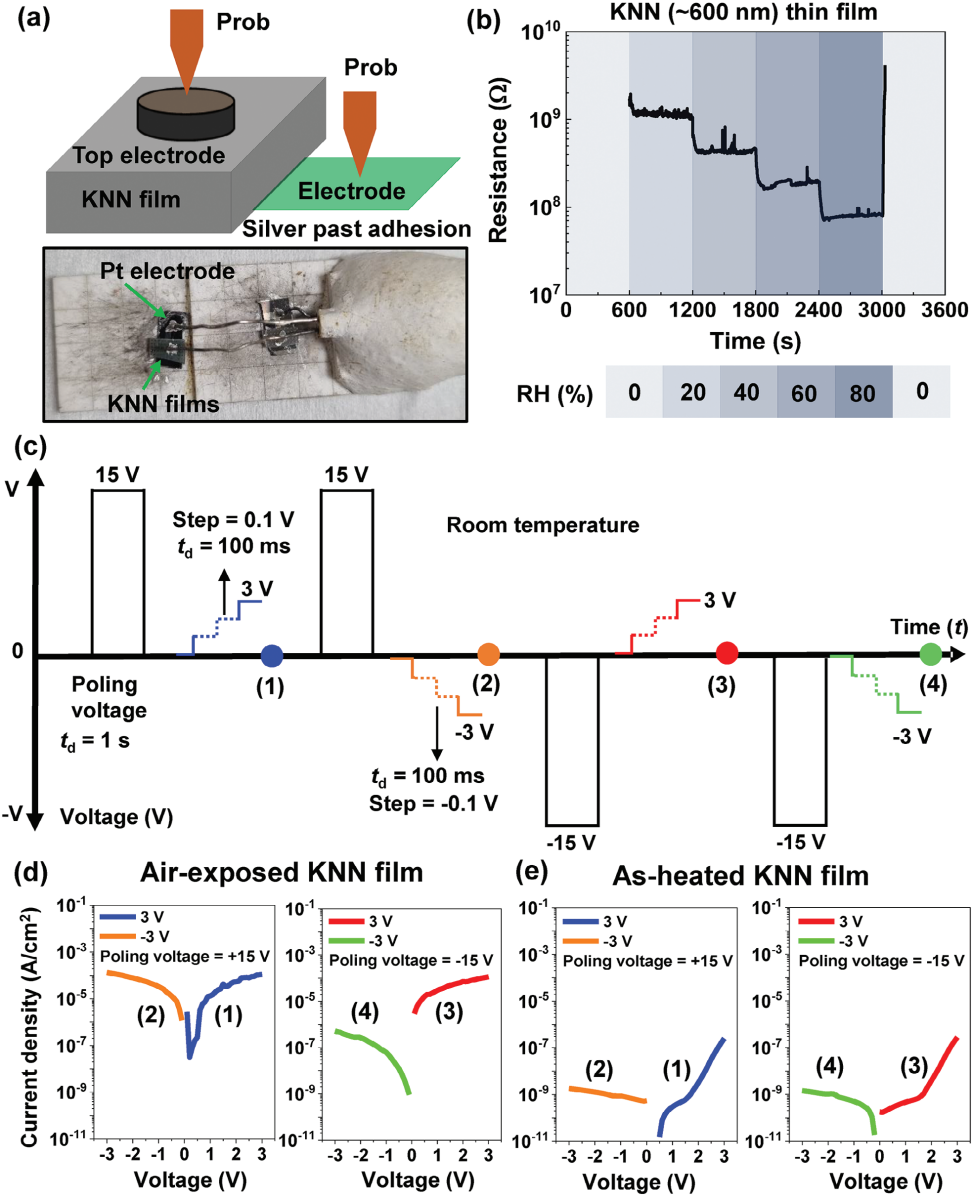
Electrical resistance and leakage current density experiments of the 600 nm‐thick KNN films. a) Schematic diagram and the optical photograph of an electrical resistance experiment for the 600 nm‐thick KNN sample. b) Resistive responses of 600 nm‐thick KNN (≈600 nm) films, a stepwise decrease in the electrical resistance of KNN sensors was observed in the air‐exposed KNN (≈600 nm) films. c–e) Leakage current results of the KNN (≈600 nm) films. (c) Schematic diagram of the leakage current obtained at the applied voltage of 3 and −3 V. At room temperature, the poling voltage was first set to 15 V and then −15 V, the step size of the increasing voltage was 0.1 V. The leakage current density data of the KNN (≈600 nm) films were acquired both in the (d) air‐exposed and (e) as‐heated state.

To gain more insight into the time‐dependent polarization retention loss of nano‐columnar KNN films under ambient air environments, we investigated the electrical characteristics of our KNN films in various humidity conditions, as shown in **Figure** **4**a. For systematic analysis of a humidity effect on the electrical resistance of the KNN films, the degree of relative humidity (RH) was precisely controlled in the range of 0 to 80% with an RH interval of 20%. Herein, the KNN samples were treated in the humidity situations for a time duration of 600 s. We first note that thicker (≈600 nm) KNN films with nanopillar structures were too insulating to measure the electrical resistance in the beginning stage (RH = 0%) due to the high film thickness. As the 600‐nm‐thick KNN films were sequentially exposed to humidity circumstances with the increasing RH, the corresponding resistance was reduced from 1.2 × 10^9^ (RH = 20%) to 8.1 × 10^7^ Ω (RH = 80%) step by step (Figure 4b). When the humidity level decreased to RH = 0%, the electrical resistance of the thicker KNN films abruptly increased close to the initial value. By contrast, as 35‐nm‐thick KNN films were exposed to the humidity environments, the electrical resistance slightly decreased in the range of 1.4 × 10^11^ (RH = 0%) to 3.7 × 10^10^ Ω (RH = 80%) with the increasing RH (Figure S9, Supporting Information). We stress that a ratio of electrical resistance between RH = 20 and 80% was roughly 1.5 × 10^1^ and 3.0 × 10° for 600‐ and 35‐nm‐thick KNN films, respectively. Considering that the RH‐dependent resistance ratio in the thicker KNN films was 5 times higher than that in the thinner KNN films, the nanopillar KNN films were more vulnerable to humidity than the planar KNN films.

To further check the electrical responses of KNN films to moisture in air relying on nanoscale crystal structures, we first prepared two different treated states (i.e., air‐exposed and as‐heated states) of 35‐ and 600‐nm‐thick KNN films. To accomplish the air‐exposed and as‐heated states, the as‐grown KNN films were treated in a high humidity condition (RH > 80%) for a day (24 h), and the treated KNN samples were heated at 550 °C for 15 min, respectively. Next, we obtained poling polarity‐dependent current density (*J*)‐voltage (*V*) curves of the air‐exposed and as‐heated KNN films at room temperature in accordance with pulsed sequences, as displayed in Figure 4c. To characterize the *J*‐*V* behaviors, we pre‐poled the KNN films by applying positive poling pulses with the amplitude (*V*_positive_) of +15 V and the width (*t*_d_) of 1 s (marked by a black color in the pulse train of Figure 4c). Then, we applied uphill step‐like positive voltage pulses with *V*_positive_ of +3 V (marked by a blue color in the pulse train of Figure 4c). Note that the amplitude (i.e., *V*_positive_ = +3 V) of the positive voltage pulses was smaller than the coercive voltages of the 600‐nm‐thick KNN films and thereby, we could simply evaluate leakage current contributions to the *J*‐*V* characteristics by minimizing the contributions of FE switching currents to the dc transport properties.^[^ ^47^ ^]^ Here, a total of 30 data points were acquired on the positive voltage sides of the measured *J*‐*V* curves with the step increment of 0.1 V and *t*_d_ of 100 ms (indicated by (1) in Figure 4c–e). Likewise, after positive pre‐poling, downhill step‐like negative voltage pulses with *V*_negative_ of −3 V (marked by an orange color in the pulse train of Figure 4c). The step decrement and *t*_d_ of the downhill negative voltage pulses were −0.1 V and 100 ms, respectively. The obtained *J*‐*V* characteristics were plotted on the negative voltage sides indicated by (2) in Figure 4c–e. In the same manner, *J*‐*V* curves after negative pre‐poling were also taken, and the obtained transport results by application of uphill positive (marked by a red color in the pulse train of Figure 4c) and downhill negative (marked by a green color in the pulse train of Figure 4c) step voltages were indicated by (3) and (4) in Figure 4c–e, respectively.

Thicker (≈600 nm) KNN films were more sensitive to humidity than thinner (≈35 nm) KNN films and hence, a humidity‐driven increase of electric conductivity was found resulting in electrical leakage. Figure 4d,e showed the measured *J*‐*V* characteristics of the air‐exposed and as‐heated 600‐nm‐thick KNN films, respectively. In the air‐exposed state, the measured current density approximately ranged from 3.2 × 10^−8^–1.1 × 10^−4^ A cm^−2^ (Figure 4d), whereas it became 3 orders of magnitude smaller (i.e., an increase in electric conductance) after thermal heat treatments (Figure 4e). However, for the thinner (≈35 nm) KNN film, the range (1.5 × 10^−11^–8.5 × 10^−9^ A cm^−2^) of current density between both the as‐exposed and as‐heated states was similar with no substantial difference (Figure S10, Supporting Information). It was also worthwhile to note that the electrical leakage of dielectric oxides under humidity environments was previously attributed to the introduction of hydrogen ions (i.e., protonation) separated from water molecules in the air into oxide materials.^[^ ^39^ ^]^

**Figure 5:**
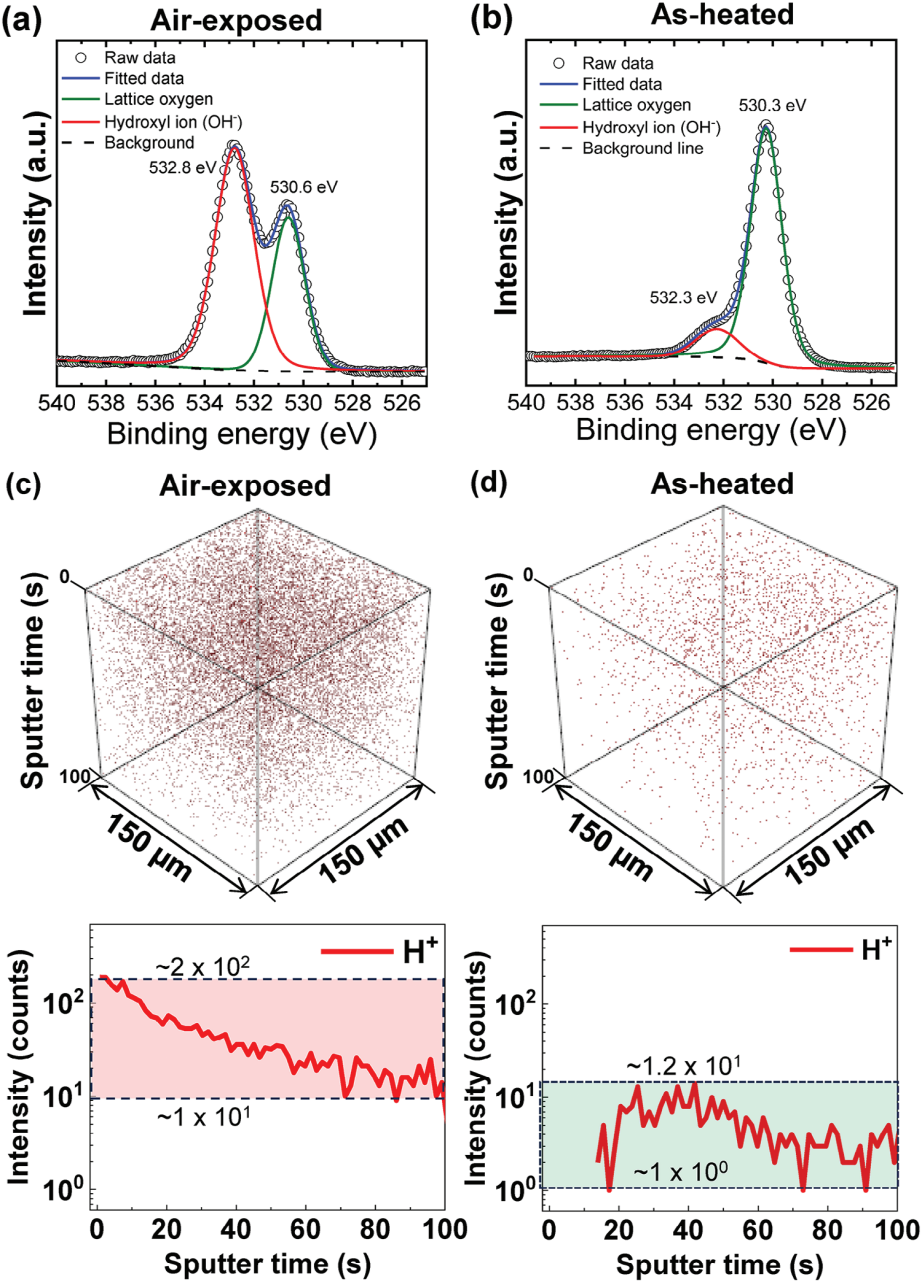
X‐ray photoelectron spectroscopy (XPS) spectra at the O 1s of the KNN (≈600 nm) grown on SrTiO_3_ substrates for a) air‐exposed and b) as‐heated. The time‐of‐flight secondary ion mass spectrometry (TOF‐SIMS) measurements. c, d) The TOF‐SIMS 3D rendering maps of H^+^ signals and the corresponding in‐depth scanning signal in the air‐exposed (for 240 h) epitaxial KNN (≈600 nm)/LSMO (≈35 nm) hetero‐bilayer thin films. The amplitude of the scanning signals from 0 to 100 sputter time (s) in the air‐exposed and as‐heated case were highlighted by the red and green rectangles, respectively. After thermal heat treatment, the amplitude of the scanning signal reduced 3 times the KNN film in the air‐exposed state.

Humidity‐driven polarization retention loss and electrical leakage in nanopillar KNN (≈600 nm) films originated from protonation via diffusion of hydrogen ions created by water dissociation in ambient air. To elucidate possible origins of the observed results microscopically, we performed x‐ray photoelectron spectroscopy (XPS) and time‐of‐flight secondary ion mass spectrometry (TOF‐SIMS) measurements of the air‐exposed (**Figure** **5**a for XPS and Figure 5c for TOF‐SIMS) and as‐heated (Figure 5b for XPS and Figure 5d for TOF‐SIMS) states of the 600‐nm‐thick KNN films. The XPS spectra at the O 1s of the air‐exposed KNN films revealed that a high‐intensity XPS peak at 532.8 eV corresponding to chemisorbed hydroxyl ions (OH^−^) was detected in the vicinity of the XPS peak of perovskite lattice oxygens (≈530.6 eV) (Figure 5a). It was energetically preferred that water molecules in air were dissociated to positively charged H^+^ (i.e., protons) and negatively charged OH^−^ ions at oxygen vacancy defects in dielectric oxides.^[^ ^37^ ^]^ Then, the produced OH^−^ ions were adsorbed at the surface oxygen vacancy sites with effective positive charges and the split protons would be introduced into the dielectric materials (i.e., protonation).^[^ ^28^, ^37^ ^]^ It was also worthwhile to note that the polarization retention loss induced by protonation was highly noticeable under acid circumstances created by acetic acid donating hydrogen ions easily (Figure S11, Supporting Information). Meanwhile, no sizeable reduction of FE polarization was observed in the measurement time limit (360 min), when our KNN films were exposed to base environments (i.e., no proton source) generated by ethanol solutions (Figure S11, Supporting Information). Additionally, the following thermal annealing (i.e., the as‐heated state) of the protonated KNN films would enable the surface‐adsorbed OH^−^ ions to be desorbed (the decrease of XPS peak intensity of chemisorbed hydroxyl ions in Figure 5b) with removal of the diffused hydrogen ions (i.e., deprotonation).

Humidity‐induced protonation in the air‐exposed states (Figure 5c) and the thermally activated deprotonation in the as‐heated states (Figure 5d) were directly evidenced in nano‐columnar KNN (≈600 nm) films. To achieve this, we visualized the amount of the dissociated hydrogen ions from water molecules from the film surfaces (*t*_sputtering_ = 0 s) inward the thin films (*t*_sputtering_ = 100 s) using the TOF‐SIMS technique. It was evident that the averaged contents (≈4.7 × 10^1^) of counted protons in the air‐exposed films were an order of magnitude higher than those (≈5.0 × 10^0^) of counted protons in the as‐heated films indicative of the deprotonation by thermal heating. On the contrary, for 2D planar KNN (≈35 nm) films, the mean value of the counted hydrogen ions before and after heat treatments were ≈1.0 × 10^1^ and ≈4.0 × 10^0^, respectively, which were mutually comparable (i.e., the corresponding ratio ≈2.5) (Figure S12, Supporting Information). Because the proportion of the counted protons between the air‐exposed and as‐heated states was much larger in the thicker KNN films (≈9.4) than the thinner KNN films (≈2.5), it was highly likely that the humidity‐driven protonation was more active in the nanopillar KNN films than the planar KNN films (Figure S13, Supporting Information). Namely, in the nano‐columnar KNN films, the adsorption of hydroxyl ions separated from water molecules and the following protonation would be more frequent thanks to the increasing surface‐to‐volume ratio^[^ ^48^, ^49^ ^]^ and/or many vacancy defects arising from the disorder at nanopillar grain boundaries.^[^ ^43^ ^]^

**Figure 6:**
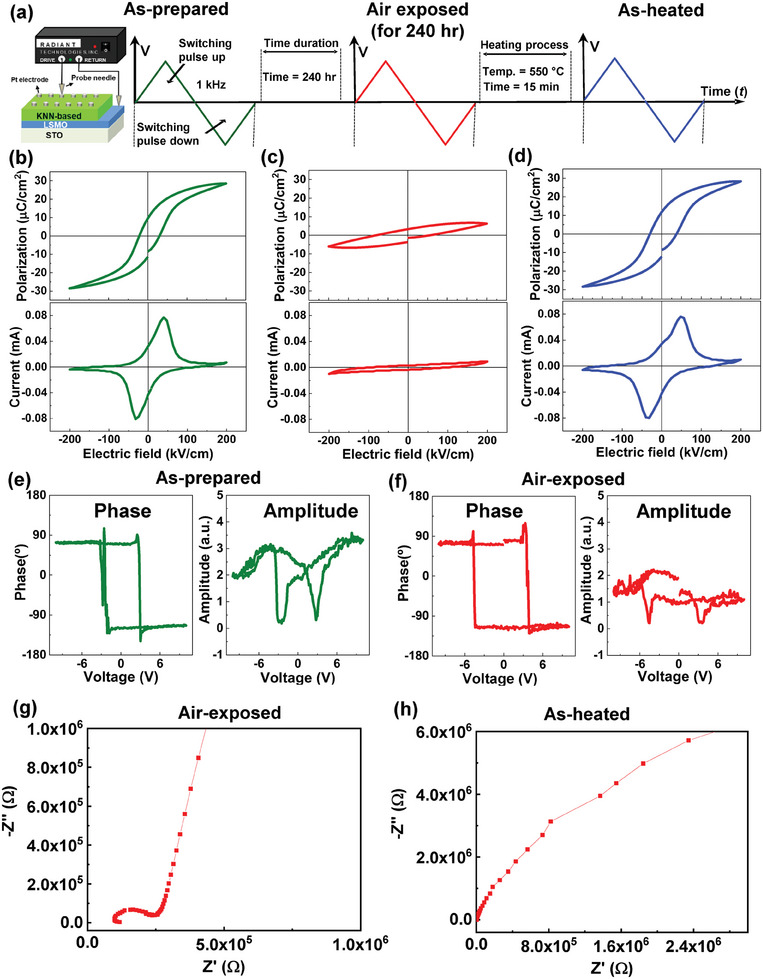
a) The graphical illustration of the epitaxial KNN (≈600 nm)/LSMO (≈35 nm) hetero‐bilayer thin films for the *P*‐*E* hysteresis and *I*‐*E* loops measurements in the as‐prepared (green), air‐exposed (red), and as‐heated (blue) states. b–d) The *P*‐*E* hysteresis loops and the corresponding *I*‐*E* loops of the KNN (≈600 nm)/LSMO (≈35 nm) hetero‐bilayer thin films in each step of as‐prepared, air‐exposed, and as‐heated states. The PFM phase and amplitude signals of the KNN (≈600 nm)/LSMO (≈35 nm) hetero‐bilayer thin films in the e) as‐prepared and f) air‐exposed states. The butterfly‐shaped PFM amplitude responses were reduced in the air‐exposed KNN sample compared with that in the as‐prepared sample showing that the polarization response was suppressed in the air‐exposed sample. Impedance spectroscopy of the g) air‐exposed and the h) as‐heated KNN (≈600 nm) films.

To understand the underlying mechanisms of protonation‐driven polarization retention failure in nano‐columnar KNN films, we examined reversibility between protonated and deprotonated states induced by air exposure and thermal annealing, respectively. As shown in **Figure** **6**a, we repetitively measured 3 different states of both *P*‐*E* hysteresis loops and FE switching currents in the as‐prepared (marked by green color in Figure 6b), air‐exposed (exposed to open air for 10 days (240 h), marked by a red color in Figure 6c), and as‐heated (heated at 550 °C for 15 min, marked by a blue color in Figure 6d) KNN (≈600 nm) films. Intriguingly, the common FE hysteresis loops/switching current curves of the as‐grown KNN films were suppressed by protonation via air exposure, and the compressed hysteresis loops/switching current curves in the protonated KNN films were restored by thermally driven deprotonation. Such a reversible control of FE hysteresis by alternating protonation and deprotonation was also reproducible (Figure S14, Supporting Information).

It was further interesting that the piezoelectric hysteresis loops obtained by piezoelectric force microscopy (PFM) were different for the as‐prepared (Figure 6e) and air‐exposed (Figure 6f) KNN (≈600 nm) films. For both the as‐prepared and air‐exposed (i.e., protonated) states, the PFM phase signals exhibited robust 180° phase difference arising from up/down polarization, although the averaged coercive voltages (≈4.0 V) of the protonated KNN films slightly increased compared with the averaged coercive voltages (≈2.7 V) of the as‐prepared KNN films. On the other hand, it was evident that the PFM amplitude of the protonated KNN films was distinctly reduced compared with the as‐prepared KNN films leading to piezoelectric loops with a suppressed butterfly shape. It was previously reported that the increasing coercive voltages and the decreasing piezoelectric amplitude were attributed to the reduction of switchable polarization by pinning of charged FE domain walls.^[^ ^29^, ^31^ ^]^ Considering that positively charged hydrogen ions were highly mobile owing to the smallest ionic radius and the lightest ionic mass,^[^ ^38^ ^]^ the hydrogen ions produced by water splitting in the nanopillar KNN films should be readily introduced into the films. The diffused hydrogen ions would be accumulated to effectively screen negative polarization bound charges at charged domain walls with tail‐to‐tail configurations^[^ ^28^, ^47^, ^50^, ^51^ ^]^ and then, the charged domain walls screened by proton accumulation were electrostatically stable with domain wall pinning (i.e., freezing switchable polarization) in the protonated KNN films (Figure S15, Supporting Information).

Electrical conducting channels were additionally formed in protonated KNN films through the trapping of hydrogen ions at the charged FE domain wall inducing polarization retention loss by domain wall pinning. The Cole‐Cole plots of the air‐exposed (Figure 6g) and as‐heated (Figure 6h) KNN films obtained by impedance spectroscopy showed that the protonated and deprotonated films exhibited double‐semicircle curves and a single semicircle arc, respectively. It was likely that vertical boundaries between columnar grains in nanopillar KNN films contained a large variety of disorders (e.g., charged vacancy defects and dislocations) and thus, the defect‐rich nano‐columnar grain boundaries served as conducting paths for electric leakage currents producing semicircle behaviors in the impedance data.^[^ ^43^, ^52^ ^]^ When the nano‐columnar KNN films were protonated by air exposure, positive hydrogen ions were accumulated at negatively charged domain walls for effective screening resulting in domain wall pinning, as aforementioned. As a direct product of the proton trapping at charged domain walls, another pathway for electrical conduction would be locally generated with the appearance of extra semicircle arcs in the Cole‐Cole plots,^[^ ^34^ ^]^ which was reminiscent of electric conduction at charged FE domain walls.^[^ ^53^, ^54^ ^]^ In addition, deprotonation by thermal annealing would enable the trapped hydrogen ions at charged domain walls to be de‐trapped, which resulted in domain wall depinning and the linked restoration of polarization retention capability. It was therefore plausible that the double‐semicircle characteristics in Cole‐Cole plots of the protonated (i.e., air‐exposed) KNN films returned to the single semicircle behaviors in the Cole‐Cole plots of the deprotonated (i.e., as‐heated) KNN films (Figure S15, Supporting Information).

## 3 Conclusion

In summary, we comprehensively characterized polarization retention properties of epitaxial lead‐free KNN thin films exhibiting two different geometries of nanoscale lattice structures by employing various experimental techniques. It was clear‐cut that the time‐dependent polarization retention behaviors under humidity environments quite varied relying on the geometrical lattice structures of the as‐grown KNN films. For thicker KNN films with nanopillar structures, progressive depletion of switchable polarization was found with suppression of FE hysteresis loops, whereas there was no sizeable retention loss in thinner KNN films with 2‐D planar structures. It was also corroborated that the polarization retention failure in the nano‐columnar KNN films was mainly associated with protonation via diffusion of hydrogen ions induced by water dissociation. Additionally, the increasing surface‐to‐volume ratio in the nanopillar KNN films would render the thicker KNN films more vulnerable to moisture than the thinner KNN films. The accumulation of hydrogen ions at charged domain walls in protonated KNN films would enable domain wall pinning by proton trapping resulting in polarization retention loss and electrical leakage. Potentially, our results will be harnessed to artificially design oxide‐based advanced electro/photo/piezo‐catalysts of versatility enabling selective manipulation of water splitting‐driven hydrogen evolution reactions according to polarization direction.

## 4 Experimental Section

### Fabrication of KNN Ceramic Target and Epitaxial Thin Films—Ceramic Target Preparation

The pure K_0.55_Na_0.55_NbO_3_ (KNN) ceramic targets were synthesized using the conventional solid‐state reaction method. Here, a 10 mol% excess quantity of sodium and potassium was added to compensate for the loss of alkaline metals due to the volatility during the high‐temperature fabrication process. Initially, the grounded powders of K_2_CO_3_ (99%), Na_2_CO_3_ (99%), and Nb_2_O_5_ (99.9%) from Sigma Aldrich were weighed and mixed. The mixture of powders was put in a bottle along with zirconia grinding balls (twice the quantity of powders) and ethanol (three times the quantity of powders) and then, ball‐milled for 24 h with a speed of 200 rev min^−1^. After ball milling, the slurries of mixed powders were dried in the oven at 100 °C for 24 h. Next, we ground the as‐dried powders and then, calcined the as‐grinded powders inside a box furnace at 800 °C for 4 h was grounded. The as‐calcined powders were re‐grinded and ball‐milled again for 24 h at the same speed. After drying in the oven for 24 h, the powders were passed through the sieving process and then, we pressurized the powders to the 1‐inch‐diameter ceramic target under a pressure of 50 MPa. Sintering procedures of pellets were performed at 1040 °C for 2 h in a box furnace.

### Fabrication of KNN Ceramic Target and Epitaxial Thin Films—KNN Films Fabrication by Pulsed Laser Deposition (PLD)

The pure KNN/LSMO heterostructures were epitaxially fabricated on the SrTiO_3_ (001) substrates using the pulsed laser deposition (PLD) method. A pulsed excimer laser (KrF, wavelength of 248 nm) with a fluence of 1.2 J cm^−2^ and a repetition rate of 5 Hz was used to generate the plasma plume from ceramic targets. Before the deposition of each layer, the corresponding ceramic target was pre‐ablated with a pulsed laser for 5 min. The LSMO film was first grown at 670 °C under an oxygen partial pressure of 20 mTorr. Subsequently, the pure KNN was deposited on the LSMO/STO substrate after changing the growth temperature to 600 °C. During the deposition of the pure KNN layer, the substrate temperature and the oxygen partial pressure were maintained at 600 °C and 100 mTorr, respectively. Finally, when the deposition of the KNN films finished, in situ, postannealing of the as‐grown films was carried out at 600 °C for 1 h under ambient oxygen at 100 Torr.

### Structural Characterizations—X‐ray Diffraction (XRD) Analyses of KNN Thin Films

A lab‐based X‐ray diffractometer (wavelength λ = 1.5406 Å; monochromated Cu Kα1, D8 Advance, Bruker, Germany) was used to execute the X‐ray diffraction (XRD) measurements of the epitaxial KNN (≈35 and ≈600 nm)/LSMO bilayer films grown on the STO (001) substrates.

### Structural Characterizations—Reciprocal Space Mapping (RSM) of KNN Thin Films

The high‐resolution RSMs of the 600 nm‐thick KNN/LSMO thin films on SrTiO_3_ (001) were performed on the synchrotron 3A beamline at the Pohang Accelerator Laboratory (PAL) with a six‐circle X‐ray diffractometer. The Lab‐source XRD machine was used for measuring the RSM of the epitaxial 35 nm‐thick KNN/LSMO/SrTiO_3_ (001) thin film heterostructures. Using the data, the in‐plane and out‐of‐plane lattice parameters of the KNN and LSMO film layers were also extracted from the *H*‐ and *L*‐scan profiles.

### Structural Characterizations—Rocking Curves Measurements of KNN Thin Films

To evaluate the crystallinity of the KNN (≈35 and ≈600 nm)/LSMO bilayer films on SrTiO_3_ (001) substrates, the rocking‐curve analyses of the KNN layers were executed. The FWHM values of the epitaxial KNN layers were obtained around the (002) diffraction peaks in both KNN (≈35 and ≈600 nm)/LSMO films. These FWHM values were estimated by fitting the raw rocking‐curve data of the (002) diffraction peaks with a Gaussian‐Lorentzian function. The raw data for the KNN films and the fitted data were represented by black scatters (square scatters for the 600 nm‐thick KNN and circular scatters for the 35 nm‐thin KNN) and red lines, respectively.

### Ferroelectric and Switching Current Measurements in KNN Thin Films

To examine the electrical properties of pure KNN films, the Pt top electrodes with an area of 2.5 × 10^−4^ cm^2^ were deposited by direct current (DC) sputtering (Cressington 108, Cressington, Inc., USA). The polarization (*P*)‐electric field (*E*) hysteresis and switching current (*I*‐*V*) loops were obtained at room temperature using a ferroelectric tester (PRECISON LC, Radiant Technologies, USA).

### Polarization Retention Experiments

Epitaxial KNN/LSMO films on SrTiO_3_ substrates were used to measure the *P*‐*E* hysteresis and *I*‐*E* loops over time while keeping/exposing the KNN/LSMO films in the open‐air environment for the protonation process through the water molecules dissociations. The measurement sequence in the retention experiment is shown in the schematic diagram, where the *P*‐*E* hysteresis and *I*‐*E* loops were measured at a time interval marked by a red circle in Figure S4, Supporting Information. For the electrical measurements, a triangular pulse with an amplitude of 8 V and a frequency of 1 kHz was used for hysteresis loop measurements. After exposing the films continuously for 10 days, the KNN/LSMO films were thermally heated in the box furnace at 550 °C for 15 min for the deprotonation of the nano‐columnar KNN films. After the thermal heat treatment, we re‐measured the *P*‐*E* and *I*‐*E* loops of the as‐heated KNN to identify the restored polarization in our samples.

### X‐ray Photoelectron Spectroscopy (XPS) Measurements

X‐ray photoelectron spectroscopy (XPS, Thermo Scientific, ESCA Probe) was performed to analyze the effects of humidity on air‐exposed and as‐heated films. The XPS was carried out using conventional monochromatic Al Kα radiation (hν = 1486.6  eV) and a fixed pass energy of 50.0 eV. The reference binding energy for adjusting other peaks was C 1s at 285.0 eV. The deconvolution of each level spectrum was conducted using a Gaussian‐Lorentzian function.

### Atomic Force Microscopy (AFM) Measurements

To measure the surface topography of the epitaxial KKN (≈35 and ≈600 nm)/LSMO heterostructure films on the SrTiO_3_ (001) substrates, the AFM (Nanocute with nanonavi 2station, Nano fine tech, Japan) was operated. The AFM images were obtained by using the dynamic force microscopy (DFM) tip with a tip radius <10 nm, resonance frequency of ≈320 kHz, and spring constant of ≈42 N m^−1^.

### Piezoresponse Force Microscopy (PFM) Measurements

To perform the PFM measurements of the KNN samples, a commercial AFM machine (NX10, Park Systems) with Pt/Ir coated conductive AFM tips (PPP‐EFM, Nanosensors) was used. The PFM amplitude and phase signals of the samples were measured by a lock‐in amplifier (HF2LI, Zurich Instrument). To enhance the signal‐to‐noise ratio of PFM responses, the dual‐frequency resonance tracking (DFRT) method was used.^[^ ^55^ ^]^

### Electrical Resistance Measurements Under Humidity Environments

The electrical resistance characteristics of the as‐grown KNN films under humidity environments were measured in a quartz tube (diameter, 3 cm; length, 30 cm) at room temperature. The RH levels (20 to 80 RH%) were calibrated by mixing the dry air with water vapor. Note that the humidity gas flow rate was 1000 sccm, which was controlled by a mass flow controller. The electrical conductivity of the KNN samples was measured by using a dc bias voltage of 0.5 V with a Keithley 2401 source meter, and all conductivity results were recorded by a homemade LabVIEW software.

### Time‐of‐Flight Secondary Ion Mass Spectrometry (TOF‐SIMS) Measurements

TOF‐SIMS experiments were performed with a M6 (ION‐TOF GmbH, Münster, Germany) in KBSI Busan Center by using a pulsed 30 keV Bi_1_ primary beam with a current 1.08 pA. The analyzed area used in this work is a square of 150 × 150 µm. Positive ion spectra were internally calibrated using H^+^, H_2_^+^, CH_3_^+^, C_2_H_5_^+^, O^+^, Nb^+^ peaks, and normalized to the respective secondary total ion yields. The chemical images of the analyzed area are recorded with 128 × 128‐pixel resolution during the data acquisition. The sputtered area is a square of 300 × 300 µm using a Cesium beam 500 eV for depth profile.

### Impedance Measurements

We analyzed the impedance characteristics of 600 nm‐thick KNN thin films (in the air‐exposed and as‐heated case) using a HIOKI 3522–50 analyzer. For the electrical impedance measurements, capacitor geometry was designed by sandwiching the KNN film layers between the Pt top electrodes (i.e., circular area of 2.5 × 10^−4^ cm^2^) and the bottom LSMO layer electrode.

### Scanning Transmission Electron Microscopy (STEM) Measurements

TEM samples were prepared by mechanical flat polishing to a thickness of <10 µm, followed by Ar+ ion beam milling using a PIPS II system from Gatan. The ion beam milling process began with an energy of 3.5 keV at an angle of 8°, and was subsequently reduced to 0.1 keV at an angle of 1°. ABF‐STEM images were acquired using a STEM (JEM‐ARM200F, JEOL) at 200 keV equipped with a 5th‐order probe corrector (ASCOR, CEOS GmbH) at the Materials Imaging and Analysis Center of POSTECH, Republic of Korea. The probe size for atomic‐scale imaging was ≈0.8 Å. The convergence semi‐angle was set at 28 mrad for ABF‐STEM imaging, with the inner and outer detector angles at 10 and 20 mrad, respectively, and a camera length of 10 cm. The raw ABF images were processed by stacking 10 slices using SmartAlign and applying band‐pass diffraction filtering to reduce background noise (SmartAlign and Filters Pro, HREM Research Inc., Japan).

## Conflict of Interest

The authors declare no conflict of interest.

## Author Contributions

M.S., C.W.A., N.X.D., and S.‐Y.H. equally contributed to this work. T.H.K., I.W.K., and S.‐Y.C. conceived and initiated the project. T.H.K., I.W.K., S.‐Y.C., S.‐H.B., S.D.B., S.M.Y., T.K.S., and S.C. supervised the experiments. M.S., C.W.A., N.X.D., I.W.K., and T.H.K. fabricated epitaxial ferroelectric films and characterized their physical properties. S.‐Y.H., G.‐Y.K., and S.‐Y.C. performed the scanning transmission electron microscopy and electron energy loss spectroscopy experiments of the as‐grown films. J.‐S.J. and S.‐H.B. carried out the humidity experiments of the as‐prepared films. E.‐Y.K. and S.D.B. conducted x‐ray photoemission spectroscopy experiments of the as‐deposited films. Y.K.K. and S.M.Y. performed piezoresponse force microscopy analyses of the as‐grown films. C.W.A., J.L., J.S.J., and J.‐S.B. carried out time‐of‐flight secondary ion mass spectrometry measurements of the as‐fabricated films. M.H.L., H.‐S.H., and T.K.S. synthesized ceramic targets for epitaxial film growth. M.S., S.C., and T.H.K. performed the atomic force microscopy measurements of the as‐grown films. M.S., C.W.A., N.X.D., S.‐Y.H., S.‐Y.C., I.W.K., and T.H.K. prepared the manuscript with contributions from all authors. T.H.K. directed this research.

## Supporting information



Supporting Information

## Data Availability

The data that support the findings of this study are available from the corresponding author upon reasonable request.

## References

[1] E. Fatuzzo , W. J. Merz , Ferroelectricity, North‐Holland Pub. Co., Amsterdam 1967.

[2] J. F. Scott , C. A. P. d. Araujo , Science 1989, 246, 1400. 17755995 10.1126/science.246.4936.1400

[3] J. F. Scott , Science 2007, 315, 954. 17303745 10.1126/science.1129564

[4] A. I. Khan , A. Keshavarzi , S. Datta , Nat. Electron. 2020, 3, 588.

[5] P. Chen , X. Zhong , J. A. Zorn , M. Li , Y. Sun , A. Y. Abid , C. Ren , Y. Li , X. Li , X. Ma , L.‐Q. Chen , X. Pan , Nat. Commun. 2020, 11, 1840. 32296053 10.1038/s41467-020-15616-y PMC7160157

[6] N. Balke , B. Winchester , W. Ren , Y. H. Chu , A. N. Morozovska , E. A. Eliseev , M. Huijben , R. K. Vasudevan , P. Maksymovych , J. Britson , S. Jesse , I. Kornev , R. Ramesh , L. Bellaiche , L. Q. Chen , S. V. Kalinin , Nat. Phys. 2012, 8, 81.

[7] S. Das , Y. Tang , Z. Hong , M. Gonçalves , M. McCarter , C. Klewe , K. Nguyen , F. Gómez‐Ortiz , P. Shafer , E. Arenholz , V. A. Stoica , S.‐L. Hsu , B. Wang , C. Ophus , J. F. Liu , C. T. Nelson , S. Saremi , B. M. Prasad , A. B. Mei , D. G. Schlom , J. Íñiguez , P. García‐Fernández , D. A. Muller , L. Q. Chen , J. Junquera , L. W. Martin , R. Ramesh , Nature 2019, 568, 368. 30996320 10.1038/s41586-019-1092-8

[8] D. Damjanovic , P. Muralt , N. Setter , IEEE Sens. J. 2001, 1, 191.

[9] X. Liu , K. Zhao , Y. Yang , Nano Energy 2018, 53, 622.

[10] H. Liu , J. Zhong , C. Lee , S.‐W. Lee , L. Lin , Appl. Phys. Rev. 2018, 5, 041306.

[11] X. K. Wei , N. Domingo , Y. Sun , N. Balke , R. E. Dunin‐Borkowski , J. Mayer , Adv. Energy Mater. 2022, 12, 2201199.

[12] S. W. Zhang , Z. Zhou , J. Luo , J. F. Li , Ann. Phys. 2019, 531, 1800525.

[13] B. Yang , Q. Zhang , H. Huang , H. Pan , W. Zhu , F. Meng , S. Lan , Y. Liu , B. Wei , Y. Liu , L. Yang , L. Gu , L.‐Q. Chen , C.‐W. Nan , Y.‐H. Lin , Nat. Energy 2023, 8, 956.

[14] A. Gruverman , M. Tanaka , J. Appl. Phys. 2001, 89, 1836.

[15] J. F. Scott , C. A. Araujo , H. B. Meadows , L. D. McMillan , A. Shawabkeh , J. Appl. Phys. 1989, 66, 1444.

[16] M. T. Ghoneim , M. A. Zidan , M. Y. Alnassar , A. N. Hanna , J. Kosel , K. N. Salama , M. M. Hussain , Adv. Electron. Mater. 2015, 1, 1500045.

[17] B. P. Maderic , L. E. Sanchez , S. Y. Wu , Ferroelectrics 1991, 116, 65.

[18] T. K. Song , J.‐G. Yoon , S.‐I. Kwun , Ferroelectrics 2006, 335, 61.

[19] C. W. Ahn , S. Y. Lee , H. J. Lee , A. Ullah , J. S. Bae , E. D. Jeong , J. S. Choi , B. H. Park , I. W. Kim , J. Phys. D: Appl. Phys. 2009, 42, 215304.

[20] H. J. Seog , A. Ullah , C. W. Ahn , I. W. Kim , S. Y. Lee , J. Park , H. J. Lee , S. S. Won , S.‐H. Kim , J. Korean Phys. Soc. 2018, 72, 1467.

[21] M. Abazari , E. K. Akdoğan , A. Safari , J. Appl. Phys. 2008, 103, 104106.

[22] L. Egerton , D. M. Dillon , J. Am. Ceram. Soc. 1959, 42, 438.

[23] B. Jaffe , W. R. Cook , H. Jaffe , Piezoelectric Ceramics, Academic Press, London 1971.

[24] L. Q. Cheng , K. Wang , F. Z. Yao , F. Zhu , J. F. Li , J. Am. Ceram. Soc. 2013, 96, 2693.

[25] J. Lin , Y. Zhou , Q. Lu , X. Wu , C. Lin , T. Lin , K.‐H. Xue , X. Miao , B. Sa , Z. Sun , J. Mater. Chem. A 2019, 7, 19374.

[26] Y. Kizaki , Y. Noguchi , M. Miyayama , Appl. Phys. Lett. 2006, 89, 142910.

[27] S. Farokhipoor , B. Noheda , APL Mater. 2014, 2, 056102.

[28] H. Lee , T. H. Kim , J. J. Patzner , H. Lu , J.‐W. Lee , H. Zhou , W. Chang , M. K. Mahanthappa , E. Y. Tsymbal , A. Gruverman , C.‐B. Eom , Nano Lett. 2016, 16, 2400. 26901570 10.1021/acs.nanolett.5b05188

[29] D. Zhang , D. Sando , P. Sharma , X. Cheng , F. Ji , V. Govinden , M. Weyland , V. Nagarajan , J. Seidel , Nat. Commun. 2020, 11, 349. 31953393 10.1038/s41467-019-14250-7 PMC6969134

[30] A. Chandrasekaran , D. Damjanovic , N. Setter , N. Marzari , Phys. Rev. B 2013, 88, 214116.

[31] S. M. Yang , T. H. Kim , J. G. Yoon , T. W. Noh , Adv. Funct. Mater. 2012, 22, 2310.

[32] H. Lu , X. Liu , J. D. Burton , C. W. Bark , Y. Wang , Y. Zhang , D. J. Kim , A. Stamm , P. Lukashev , D. Felker , C. M. Folkman , P. Gao , M. S. Rzchowski , X. Q. Pan , C.‐B. Eom , E. Y. Tsymbal , G. A. , Adv. Mater. 2012, 24, 1209. 22278910 10.1002/adma.201104398

[33] Y. S. Oh , L. Wang , H. Lee , W. S. Choi , T. H. Kim , Adv. Funct. Mater. 2023, 33, 2302261.

[34] N. X. Duong , J.‐S. Jang , M.‐H. Jung , J.‐S. Bae , C. W. Ahn , J. S. Jin , K. Ihm , G. Kim , S. Y. Lim , J. Lee , D. D. Dung , S. Lee , Y.‐M. Kim , S. Lee , S. M. Yang , C. Sohn , I. W. Kim , H. Y. Jeong , S.‐H. Bake , T. H. Kim , Sci. Adv. 2023, 9, eadd8328. 36827373 10.1126/sciadv.add8328 PMC9956132

[35] I. Vinogradov , S. Singh , H. Lyle , M. Paolino , A. Mandal , J. Rossmeisl , T. Cuk , Nat. Mater. 2022, 21, 88. 34725518 10.1038/s41563-021-01118-9

[36] Y. Tian , L. Wei , Q. Zhang , H. Huang , Y. Zhang , H. Zhou , F. Ma , L. Gu , S. Meng , L.‐Q. Chen , C.‐W. Nan , J. Zhang , Nat. Commun. 2018, 9, 3809. 30228308 10.1038/s41467-018-06369-w PMC6143547

[37] R. Schaub , P. Thostrup , N. Lopez , E. Lægsgaard , I. Stensgaard , J. K. Nørskov , F. Besenbacher , Phys. Rev. Lett. 2001, 87, 266104. 11800845 10.1103/PhysRevLett.87.266104

[38] R. D. Shannon , Acta Crystallogr. A 1976, 32, 751.

[39] M. Saito , T. Sakurai , S. Ito , H. Yamamura , Procedia. Eng. 2012, 36, 74.

[40] U. Aschauer , R. Pfenninger , S. M. Selbach , T. Grande , N. A. Spaldin , Phys. Rev. B 2013, 88, 054111.

[41] D. Lin , Z. Li , S. Zhang , Z. Xu , X. Yao , J. Am. Ceram. Soc. 2010, 93, 941.

[42] T. H. Kim , T. R. Paudel , R. J. Green , K. Song , H.‐S. Lee , S.‐Y. Choi , J. Irwin , B. Noesges , L. J. Brillson , M. S. Rzchowski , G. A. Sawatzky , E. Y. Tsymbal , C. B. Eom , Phys. Rev. B 2020, 101, 121105.

[43] H. Liu , H. Wu , K. P. Ong , T. Yang , P. Yang , P. K. Das , X. Chi , Y. Zhang , C. Diao , W. K. A. Wong , E. P. Chew , Y. F. Chen , C. K. I. Tan , A. Rusydi , M. B. H. Breese , D. J. Singh , L.‐Q. Chen , S. J. Pennycook , K. Yao , Science 2020, 369, 292. 32675370 10.1126/science.abb3209

[44] S. Hu , Y. Wang , C. Cazorla , J. Seidel , Chem. Mater. 2017, 29, 708.

[45] D. Bach , R. Schneider , D. Gerthsen , Microsc. Microanal. 2009, 15, 524. 19852875 10.1017/S1431927609991061

[46] J. Scott , J. Phys.: Condens. Matter 2007, 20, 021001.

[47] T. H. Kim , B. C. Jeon , T. Min , S. M. Yang , D. Lee , Y. S. Kim , S. H. Baek , W. Saenrang , C. B. Eom , T. K. Song , J.‐G. Yoon , T. W. Noh , Adv. Funct. Mater. 2012, 22, 4962.

[48] J. S. Jang , C. W. Ahn , S. S. Won , J. H. Kim , W. Choi , B.‐S. Lee , J.‐H. Yoon , H. G. Kim , J. S. Lee , J. Phys. Chem. C 2017, 121, 15063.

[49] C. W. Ahn , P. H. Borse , J. H. Kim , J. Y. Kim , J. S. Jang , C.‐R. Cho , J.‐H. Yoon , B.‐s. Lee , J.‐S. Bae , H. G. Kim , J. S. Lee , Appl. Catal. B: Environ. 2018, 224, 804.

[50] Y. Zuo , Y. A. Genenko , B.‐X. Xu , J. Appl. Phys. 2014, 116, 044109.

[51] S. H. Baek , C. M. Folkman , J. W. Park , S. Lee , C. W. Bark , T. Tybell , C. B. Eom , Adv. Mater. 2011, 23, 1621. 21472789 10.1002/adma.201003612

[52] H. Wu , S. Ning , M. Waqar , H. Liu , Y. Zhang , H.‐H. Wu , N. Li , Y. Wu , K. Yao , T. Lookman , X. Ding , J. Sun , J. Wang , S. J. Pennycook , Nat. Commun. 2021, 12, 2841. 33990584 10.1038/s41467-021-23107-x PMC8121868

[53] J. Seidel , L. W. Martin , Q. He , Q. Zhan , Y.‐H. Chu , A. Rother , M. E. Hawkridge , P. Maksymovych , P. Yu , M. Gajek , N. Balke , S. V. Kalinin , S. Gemming , F. Wang , G. Catalan , J. F. Scott , N. A. Spaldin , J. Orenstein , R. Ramesh , Nat. Mater. 2009, 8, 229. 19169247 10.1038/nmat2373

[54] T. Rojac , A. Bencan , G. Drazic , N. Sakamoto , H. Ursic , B. Jancar , G. Tavcar , M. Makarovic , J. Walker , B. Malic , D. Damjanovic , Nat. Mater. 2017, 16, 322. 27842075 10.1038/nmat4799

[55] B. J. Rodriguez , C. Callahan , S. V. Kalinin , R. Proksch , Nanotechnology 2007, 18, 475504.

