# Supplementary material for: Protonation‐Driven Polarization Retention Failure in Nano‐Columnar Lead‐Free Ferroelectric Thin Films
Source: Adv Sci (Weinh). 2024 Nov 3;11(48):2408784. doi: 10.1002/advs.202408784 (PMC11672311; doi:10.1002/advs.202408784)
Supplement: Supplementary file 1 — Supporting Information [file ADVS-11-2408784-s001.docx]

Protonation-driven Polarization Retention Failure in Nano-columnar Lead-free Ferroelectric Thin Films

*Muhammad Sheeraz^a)^, Chang Won Ahn^a)^, Nguyen Xuan Duong^a)^, Soo-Yoon Hwang^a)^, Ji-Soo Jang, Eun-Young Kim, Yoon Ki Kim, Jaeyeong Lee, Jong Sung Jin, Jong-Seong Bae, Myang Hwan Lee, Hyoung-Su Han, Gi-Yeop Kim, Shinuk Cho, Tae Kwon Song, Sang Mo Yang, Sang Don Bu, Seung-Hyub Baek, Si-Young Choi^*^, Ill Won Kim^*^, and Tae Heon Kim^*^*

M. Sheeraz, C. W. Ahn, N. X. Duong, S. Cho, I. W. Kim, T. H. Kim

Department of Physics and Energy Harvest-Storage Research Center (EHSRC)

University of Ulsan

Ulsan 44610, Republic of Korea

E-mail: thkim79@ulsan.ac.kr; kimiw@ulsan.ac.kr

S.-Y. Hwang, G.-Y. Kim, S.-Y. Choi

Department of Materials Science and Engineering

Pohang University of Science and Technology

Pohang 37673, Republic of Korea

E-mail: youngchoi@postech.ac.kr

J.-S. Jang, S.-H. Baek, T. H. Kim

Electronic Materials Research Center

Korea Institute of Science and Technology

Seoul 02792, Republic of Korea

E-mail: thkim79@kist.re.kr

E.-Y. Kim, S. D. Bu

Department of Physics, Research Institute of Physics and Chemistry

Jeonbuk National University

Jeonju 54896, Republic of Korea

Y. K. Kim, S. M. Yang

Department of Physics

Sogang University

Seoul 04107, Republic of Korea

J. Lee, J. S. Jin, J.-S. Bae

Busan Center

Korea Basic Science Institute (KBSI)

Busan 46742, Republic of Korea

M. H. Lee, T. K. Song

School of Materials Science and Engineering

Changwon National University

Gyeongnam, 51140, Republic of Korea

H.-S. Han

School of Materials Science and Engineering

University of Ulsan

Ulsan 44776, Republic of Korea

S.-H. Baek

Division of Nano & Information Technology, KIST School

University of Science and Technology

Seoul 02792, Republic of Korea

S.-Y. Choi

Center for Van der Waals Quantum Solids

Institute for Basic Science

Pohang 37673, Republic of Korea

S.-Y. Choi

Department of Semiconductor Engineering

Pohang University of Science and Technology

Pohang 37673, Republic of Korea

^a)^ Contributions: M. Sheeraz, C. W. Ahn, N. X. Duong, and S.-Y. Hwang contributed equally to this work.

**Keywords:** ferroelectric, (K,Na)NbO_3_, thin film, polarization retention loss, epitaxy

**Protonation-driven** **polarization retention loss in lead-free K_0.5_Na_0.5_NbO_3_ (KNN) films**

**Figure S1.** A possible mechanism of the observed polarization retention loss behavior in K_0.5_Na_0.5_NbO_3_ (KNN) thin films in the as-prepared and air-exposed. The polarization retention loss behavior in K_0.5_Na_0.5_NbO_3_ (KNN) thin films is largely attributed to the effective screening of polarization-bound charges and/or pinning of charged domain walls by the accumulation of mobile hydrogen ions dissociated from water molecules in air.^[1-4]^

**Epitaxial KNN/La_0.7_Sr_0.3_MnO_3_ (LSMO) thin film heterostructures on SrTiO_3_ (001) substrates**

Figure S2a showed the XRD *θ*-2*θ* scans of the thinner (~35 nm) KNN/LSMO thin films on SrTiO_3_ (001) substrates. It was evident that the thinner KNN films were grown on the LSMO/ SrTiO_3_ with no secondary phase. The full-width half maximum (FWHM) values obtained from rocking curves of (002) diffraction peak of thinner KNN were ~0.30° for thinner (~35 nm) films [the inset of Figure S2a]. The AFM image exhibited a rough surface with a high root mean square (rms) roughness of 16.1 nm due to partial strain relaxation of the film [Figure S2b]. In the reciprocal space mapping (RSM) results of the 35 nm-thick KNN films, it was evident that all H values of KNN film layers and LSMO layers were similar to the (103) diffraction peak of the SrTiO_3_ substrates with little variation in the KNN film peak. This indicated that both the KNN and LSMO films were completely under in-plane compressive strain with respect to the underlying SrTiO_3_ substrates, leading to coherent growth. In the as-strain state, the in-plane and out-of-plane lattice constants of the KNN (LSMO) films were measured to *a*_KNN_ = 3.905 (*a*_LSMO_ = 3.905) and *c*_KNN_ = 3.993 (*c*_LSMO_ = 3.899) Å, respectively. Considering that the pseudocubic in-plane (*a*_pc_) and out-of-plane (*c*_pc_) lattice constants of bulk KNN were 3.932 and 3.969 Å,^[5]^ respectively, the 35 nm-thick KNN films were strained along the in-plane direction. The annular bright field (ABF) scanning transmission electron microscopy (STEM) analyses, the 35 nm-thick KNN films demonstrated the formation of 2D planar structural geometry under the influence of the compressive strain, similar to the conventional growth of the heteroepitaxial thin films [Figure S2d].

**Figure S2.** Epitaxial KNN/LSMO thin film heterostructures grown on SrTiO_3_ (001) substrates. a) The XRD analyses of the as-grown KNN (∼35 nm)/LSMO (~15 nm) hetero-bilayer thin films. The rocking-curve measurement [in the inset of the Figure S1a] of the KNN (∼35 nm)/LSMO (~15 nm) films. From the Bragg peak (002) of the thinner KNN in the rocking curve, we identified the full width at half maximum (FWHM) values were ∼0.30° for KNN (∼35 nm)/LSMO (~15 nm) thin films. b) Atomic force microscopy (AFM) topographical image of the epitaxial KNN layer in the KNN (∼35 nm)/LSMO (~15 nm) films. c) Lab source RSMs of pure KNN (~35 nm)/LSMO (~15 nm) films around the (103) Bragg peaks of SrTiO_3_ (001) substrates. d) The cross-sectional STEM image of the as-grown KNN (∼35 nm)/LSMO (~15 nm) films on the SrTiO_3_ (001) substrates. The scale bar in the STEM image is 50 nm.

**Magnified scanning transmission electron microscopy (STEM) analyses of 35 nm-thick KNN films**

To visualize the atomic-scale lattice structures of KNN films, we performed magnified cross-section STEM analyses of the 35-nm thick KNN films, as depicted in Figure S3. Similar to the conventional growth of oxide heterostructure film, we evidenced that the KNN films were epitaxially grown on LSMO/SrTiO_3_ with a single domain structure up to a thickness of ~3 nm. The KNN film layers were homogenously strained along with the LSMO layers with respect to the underlying SrTiO_3_ (001) substrates. In the magnified cross-sectional STEM images, the average width of the observed nanopillar structures was approximately 3 nm (marked by the double-sided yellow arrow in Figure S3) identical to a recent report of nanopillar structures.^[6]^


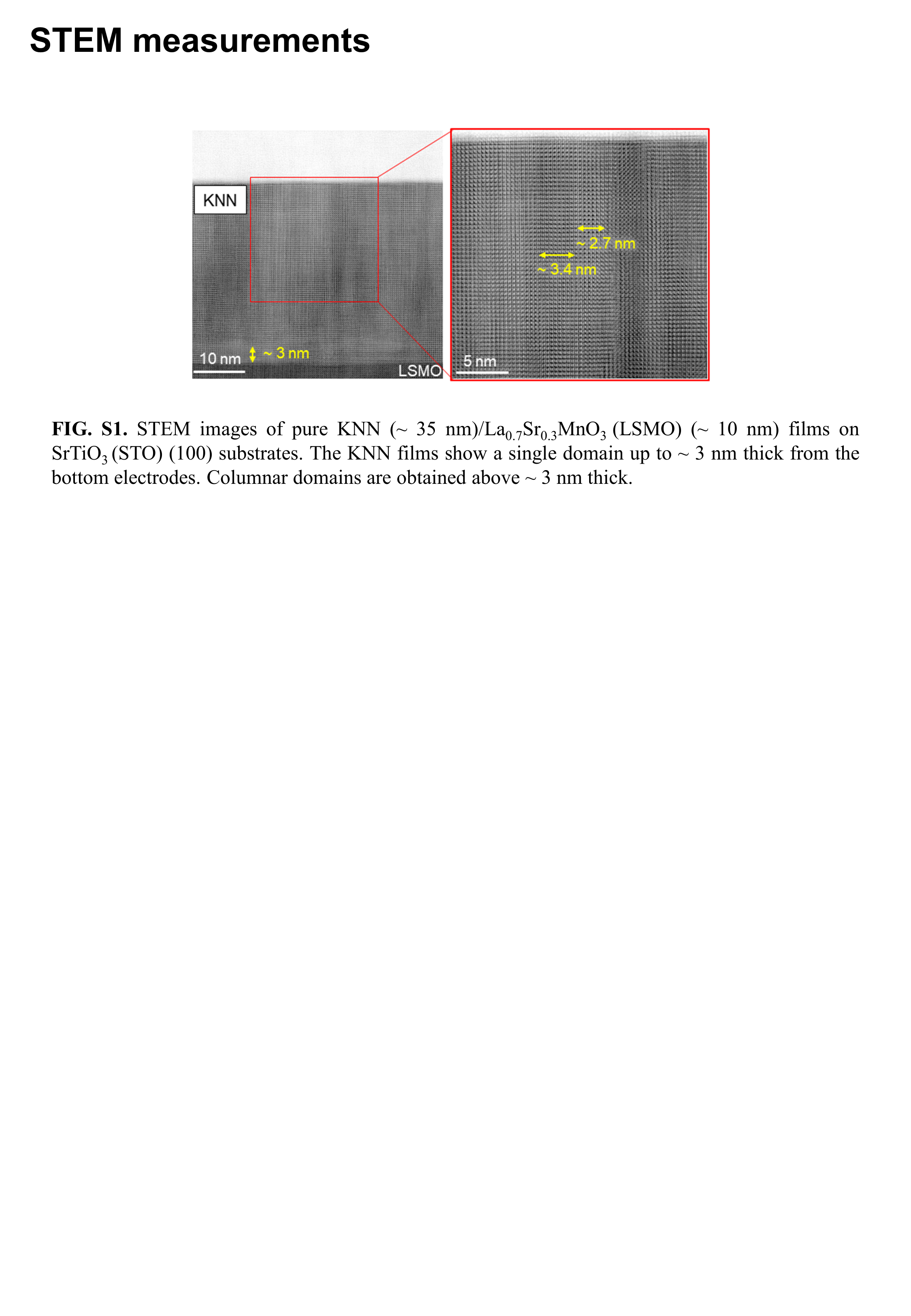


**Figure S3**. The scanning transmission electron microscopy (STEM) images of pure KNN (~35 nm)/LSMO (~15 nm) films on SrTiO_3_ (100) substrates. The KNN films show a single domain up to ~3 nm thick from the bottom electrodes. The width of the nano-pillars domains is close to ~3 nm in thickness.

**Set-up of polarization retention experiments**

To assess the polarization retention behavior of the epitaxial KNN (~35 and ~600 nm)/LSMO thin films on the SrTiO_3_ (001) substrates, we measured the polarization (*P*)-electric field (*E*) hysteresis loops of the KNN films. For evaluating the time-dependent polarization retention in the KNN thin films, we obtain the *P*-*E* hysteresis loops (i.e., at the applied bipolar triangular pulses in green color with a frequency of 1 kHz) of the air-exposed KNN films for continuously 10 days (240 h) [Figures S4a,b]. In the schematic Figure S4c, the *P*-*E* hysteresis loops were obtained over time (marked by the red circles) while the KNN film was exposed to ambient air. Next, the thin-film specimens remained in the air with no electric stress for a time duration (*t*). After a lapse of *t*, we measured *P*-*E* and *I*-*E* loops again.

**Figure S4.** a) A schematic diagram of ferroelectric hysteresis loop measurement for KNN films. b) A triangular pulse with an amplitude of 8 V and a frequency of 1 kHz was used for polarization hysteresis loop measurements. c) The measurement sequence in the retention experiment was marked by a red circle representing the time-dependent *P*-*E* hysteresis loop and *I*-*E* curve measurements of the KNN films.

**Polarization-electric field (*P*-*E*) and switching current (*I*-*E*) loops of KNN (~600 nm) films**

The *P*-*E* hysteresis and the *I*-*E* response of the thicker (~600 nm) KNN films in the as-prepared state and air-exposed in the open air over time were shown in Figure S5. Note that the thicker KNN films showed well-saturated *P*-*E* loops with a presence of imprint behavior at room temperature evident in their corresponding *P*-*E* hysteresis and *I*-*E* loops. In the as-prepared state, we extracted the maximum and remnant polarization values, it was found that the 600 nm-thick KNN film exhibits remnant polarization (*P*_rem_^+^ = 7.213 and *P*_rem_^-^ = -12.424 μC cm^-2^) and maximum polarization film (*P*_max_^+^ = 28.814 and *P*_max_^-^ = -28.814 μC cm^-2^). When the 600 nm-thick KNN samples were exposed to an open-air environment, we noticed that the maximum polarization values of the corresponding films continuously decreased where the remnant polarization values initially increased and then began to reduce after exposing the sample to open air for 5 days. The increase in remnant polarization values of the KNN films was extrinsic and could be associated with the increase in leakage current density, as noticed in the *I*-*E* loops after the exposure of the KNN films for up to five days^[7]^ (Figure 3c in the main manuscript). Finally, on the tenth day, the remnant and maximum polarization values were reduced below 5 μC cm^-2^ in the KNN films, along with a progressive decrease in leakage current density (Figure 3a,d in the main manuscript). This demonstrated that the ferroelectric polarization response in the thicker KNN (~600 nm) was highly vulnerable to humidity/moisture in the air.

**Figure S5.** The time-dependent evolution of *P*-*E* hysteresis loops and the *I*-*E* curves of the thick KNN (~600 nm) films over their exposure in the open air.

**Electric polarization of KNN (~35 nm)/LSMO (~15 nm) films on SrTiO_3_ (001) substrates over time**

The retention responses of the 35 nm-thick KNN films were also examined under the same conditions used for the thicker 600 nm-thick KNN films [Figure S6]. In contrast to KNN (~600 nm), the 35 nm-thick KNN films demonstrated nearly similar polarization loops and the switching current response consistently up to 10 days (240 h) with a minor decrease in polarization values [Figure S6a]. The thinner (~35 nm) KNN films showed *P*_rem_^+^ (*P*_rem_^-^) = 2.9 (-8.2) and *P*_max_^+^ (*P*_max_^-^) = 19.1 μC cm^-2^ (-19.3 μC cm^-2^), obtained from Figure S6b. Moreover, the thinner KNN films consistently showed an imprint behavior at room temperature evident in their corresponding *P*-*E* hysteresis and *I*-*E* loops, demonstrating that the ferroelectric polarization response in the thinner KNN (~35 nm) was not affected by humidity/moisture in the air.

**Figure S6.** Maximum polarization and remanent polarization over time for (a) KNN (~35 nm)/LSMO (~15 nm) films. *P*-*E* hysteresis loops and *I*-*E* curves of (b) KNN (~35 nm) films at the initial state, (c) KNN films after 120 h, and (d) after 240 h of air exposure at room temperature.

**Polarization-electric field (*P*-*E*) and switching current (*I*-*E*) loops of KNN (~35 nm) films**

The *P*-*E* hysteresis and *I*-*E* loops of the KNN (~35 nm) were measured at the applied electric field of 500 kV/cm using the Radiant source technologies, as demonstrated in Figure S7. It can be seen that the KNN films showed well-saturated hysteresis loops over time while exposed to an ambient air environment. Furthermore, the KNN films presented lower positive remnant and higher negative remnant polarization values employing the imprinting behavior at room temperature *P*-*E* hysteresis, further evident in their corresponding *I*-*E* loops [Figure S7].

**Figure S7**. Polarization hysteresis loops and the switching current characteristics of the thin KNN (~35 nm) films over their exposure in the open air.

**Ferroelectric polarization response of the KNN (~35 and ~600 nm) films after vacuum treatment**


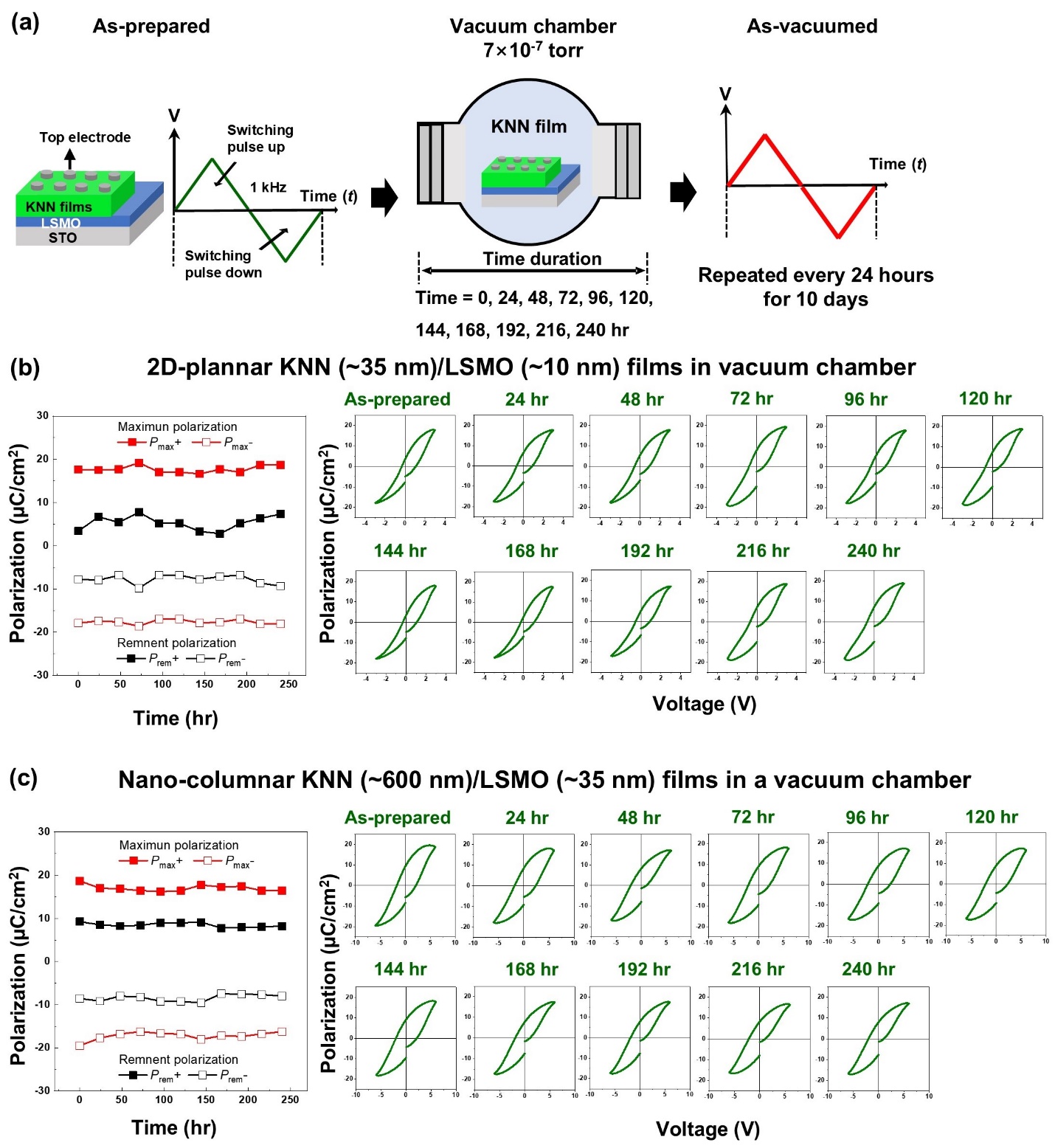


**Figure S8.** a) A graphical representation of epitaxial KNN (~35 nm and ~600 nm)/LSMO hetero-bilayer thin films illustrating the *P*-*V* hysteresis behavior in both the as-prepared (green) and vacuumed (red) states. b,c) The time-dependent evolution of ferroelectric (FE) hysteresis was carried out every 24 h for both the 35 nm and 600 nm-thick KNN films. To ensure consistency, the polarization hysteresis loops were initially measured for the as-fabricated KNN (~35 nm and 600 nm) films. The films were then placed in a vacuum chamber at a low pressure of ~7×10⁻^7^ torr. Every 24 h, we removed the KNN samples to measure the ferroelectric hysteresis loops and then re-vacuumed them, as shown in Figure S8a. This process was repeated consistently over 10 days. b) The extracted maximum (*P*_max_⁺ and *P*_max_⁻) and remnant (*P*_rem_⁺ and *P*_rem_⁻) polarization values of the epitaxial KNN (~35 nm)/LSMO (~10 nm) hetero-bilayer thin films on SrTiO_3_ substrates are shown over time (up to 10 days; 240 h). The corresponding *P*-*V* hysteresis loops for the KNN (~35 nm) thin films in the as-prepared and vacuumed states (up to 240 h) were represented in green. c) The polarization values and associated *P*-*V* hysteresis loops of the thicker KNN (~600 nm)/LSMO (~35 nm) hetero-bilayer thin films were similarly highlighted, using the same color scheme as for the thinner KNN films.

**Humidity sensing experiment of the 35 nm-thick KNN films**

To implement the protonation of the KNN films for further implications, we also evaluated the electrical resistance measurement of our KNN (~35 nm) films in the presence of the relative humidity environment.^[8]^ The schematic diagram and the optical photograph of the humidity-sensing experiment were presented in Figure S9a. A capacitor geometry with top and bottom silver electrodes was prepared to monitor the development of electrical resistance in humid conditions. In the experimental setup, the relative humidity (RH) gradually increased from 0 to 80% with a time interval of about 1000 s. For comparative analyses in the humidity sensing experiment, we used RH = 0% as the reference condition for the air atmosphere. In contrast, the KNN (~35 nm) films initially showed constant resistance without any change up to RH = 40%. Then electrical resistance decreased from 1.4 × 10^11^ to 3.7 × 10^10^ in relatively higher humidity concentrations RH = 80% [Figure S9b].

**Figure S9.** Sensing performance experiments of the KNN thinner (~35 nm) films. a) Optical photograph image of a humidity sensing experiment for the KNN sample. b) Resistive responses of thinner (~35 nm) films remained constant up to 1800 s and then began to decrease slightly. A stepwise decrease in the electrical resistance of KNN sensors was observed in air-exposed KNN (~35 nm) films.

**Leakage current density (*J*-*V*) of the KNN (~35 nm) films**

We also performed leakage current density (*J*-*V*) measurements of the air-exposed (i.e., KNN films left in an ambient air environment for 240 h) and as-heated (i.e., thermally heated at 550 °C for 15 min) KNN (~35 nm) films. The measurement conditions of the *J*-*V* data are shown in the schematic Figure S10a. To characterize the *J*-*V* curves, we first pre-poled the KNN films by applying positive poling pulses with an amplitude of +4 V and a width of 1 s, as marked in black in Figure S10a. Then, we applied uphill step-like positive voltage pulses with an amplitude of +1 V, as marked in blue in Figure 10Sa. The step increment and *t*_d_ of the downhill negative voltage pulses were -0.1 V and 100 ms, respectively. Similarly, after positive pre-poling, downhill step-like negative voltage pulses with *V*_negative_ of -1 V [marked by an orange color in the pulse train of Figure 10Sa]. The step decrement and *t*_d_ of the downhill negative voltage pulses were -0.1 V and 100 ms, respectively. The results showed that the air-exposed film exhibited nearly the same current density as the as-heated KNN films, as presented in Figure S10b,c. The same steps were repeated to obtain the *J*-*V* curves at positive (+1 V) and negative (-1 V) voltage pulses in the presence of the negative poling voltage of -4 V.

**Figure S10.** Leakage current results of the KNN (~35 nm) films. a) Schematic diagram of the leakage current obtained at the applied voltage of 1 and -1V. At room temperature, the poling voltage was first set to 4 V and then -4 V while the frequency was kept consistent to 1 kHz. The step size of the increasing voltage was 0.1 V. The leakage current data of the KNN (~35 nm) films were obtained both in the (b) air-exposed and (c) as-heated states.

**Evolution** **of ferroelectric hysteresis loops of 600 nm-thick KNN films exposed in different environments**

We monitored the evolution of ferroelectric hysteresis loops in thicker KNN films exposed to three different pH environments over varying time intervals: (i) weak acid [acetic acid (CH_3_COOH)], (ii) neutral [water vapor (H_2_O)], and (iii) base [ethanol (C_2_H_5_OH)]. Initially, the ferroelectric hysteresis loops of the as-prepared 600 nm-thick KNN films were measured. The films were then placed above a tray containing the chemical solutions in vacuumed desiccators (~380 torr) to avoid direct contact, and the desiccators were sealed for specific time intervals (0, 10, 60, 120, 240, and 360 min) with ~80% humidity [Figure S11a]. Time-dependent ferroelectric hysteresis loop measurements revealed that the rate of polarization retention loss induced by protonation varied significantly with the chemical environment [Figures S11b-d]. Rapid polarization failure occurred in the acidic environment (~10 min), while no significant change was observed in the base environment within the time interval of 360 min. In the neutral water environment, retention loss occurred took place (< 360 min) between the acid and base cases. This followed that polarization retention characteristics were vulnerable to the pH levels of chemical environments. Particularly, in the acidic environment, where protons (i.e., hydrogen ions) were easily donated and diffused into KNN films. In contrast, the diffusion of hydrogen ions was blocked in the base environment due to the lack of available protons.


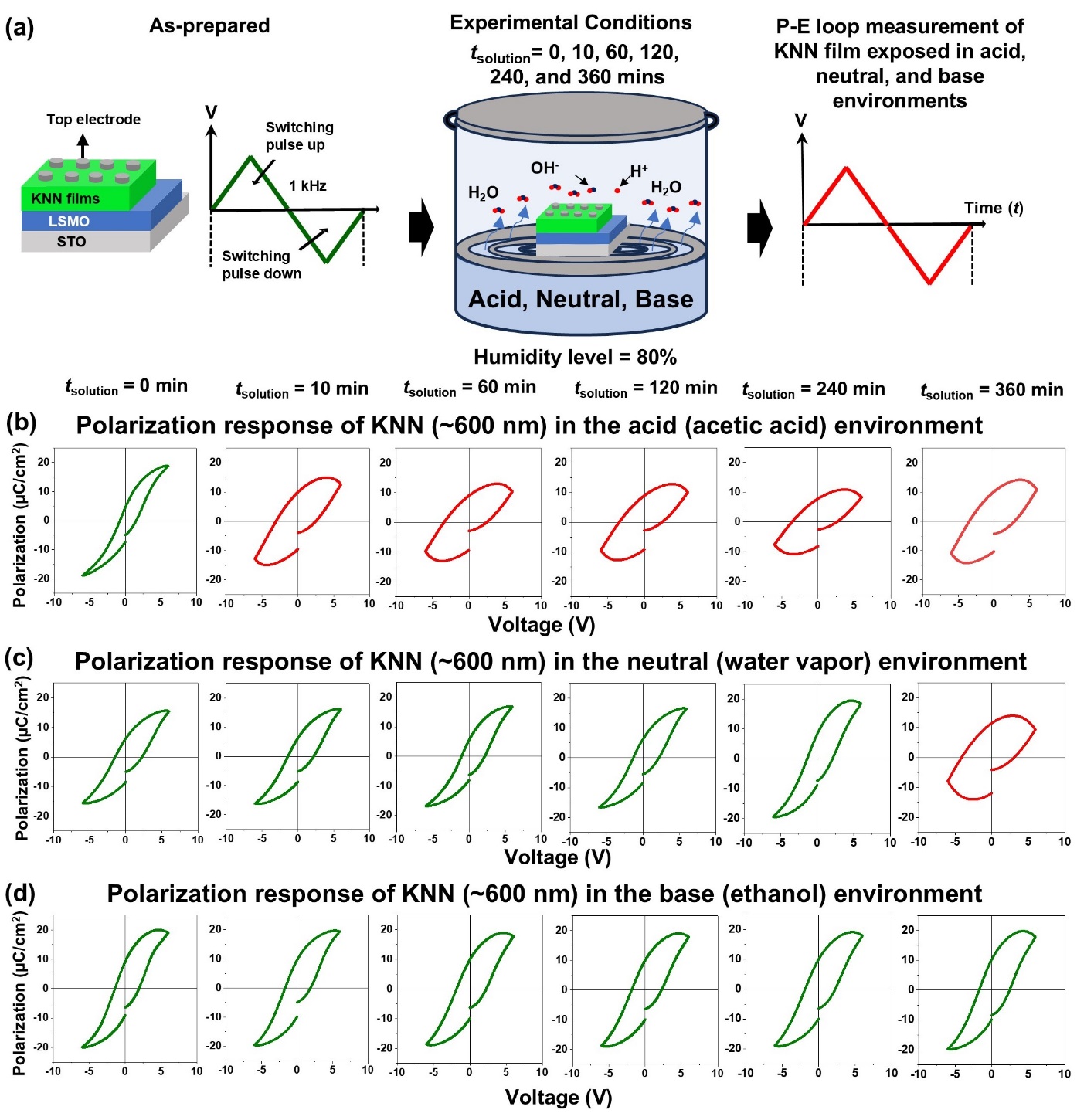


**Figure S11.** a) The graphical illustration of the epitaxial KNN (~600 nm)/LSMO hetero-bilayer thin films for the *P*-*V* hysteresis in the as-prepared and the specific environment (i.e., from 10 to 360 min) exposed states. Polarization response of KNN (~600 nm) in the (b) acetic acid (c) water vapors (d) ethanol environment with varying time from 10 to 360 min. The polarization hysteresis loops of the epitaxial KNN (∼600 nm) films quickly changed when exposed to only 10 min and then continuously showed lossy hysteretic characteristics up to 360 min. In the case of water exposure, ferroelectric polarization changes to lossy loops upon exposure to 360 min. The *P*-*V* hysteresis loop of the KNN films nearly remained the same up to 360 min.

**Time-of-flight secondary ion mass spectrometry (TOF-SIMS) 3D rendering maps of H^+^ signals**

To verify the presence of the hydrogen (H^+^) ion in KNN (~35 nm) films, we also performed TOF-SIMS analyses both in the air-exposed and as-heated states. Water molecules in the air were energetically influenced to dissociate into positively charged H^+^ ions (protons) and negatively charged OH^-^ ions at oxygen vacancy defects in dielectric oxides.^[3,8]^ The resulting OH^-^ ions were then adsorbed onto the surface oxygen vacancy sites with effective positive charges, while the protons were introduced into the dielectric materials. In the air-exposed KNN films, a minor amount of hydrogen (H^+^) ions was noticed, present homogeneously in the KNN films after the dissociation of water molecules as shown in the 3D rendering maps of H^+^ signals and the corresponding in-depth scanning signal from top to bottom [Figure S12a]. The quantity of the hydrogen ions incorporated in the films was reduced from the ~2.5 × 10^1^ (~2.5 × 10^0^) to ~1.1 × 10^1^ (~1 × 10^0^) counts for the sputtering time of 0 s (100 s), respectively [Figure S12a,b]. Even though the H^+^ ions signals were detected in the air-exposed KNN (~35 nm) films, the quantity of the H^+^ was too low thus not affecting the net polarization response in the air-exposed case. This suggested that the incorporation/diffusion of hydrogen ions via water dissociation was closely related to the observed retention failure in thicker (~600 nm) KNN compared to 35 nm-thick KNN films.

**Figure S12.** TOF-SIMS 3D rendering maps of H^+^ signals in the KNN (~35 nm)/LSMO (~15 nm) films (a) after a retention time and (b) after re-annealing (scale: 150 µm × 150 µm). Time of flight secondary ion mass spectrometry of KNN (~35 nm)/LSMO (~15 nm) films (c) air-exposed and (d) as-heated case.

**Schematic figure of the polarization response in the 35 nm-thick KNN film**


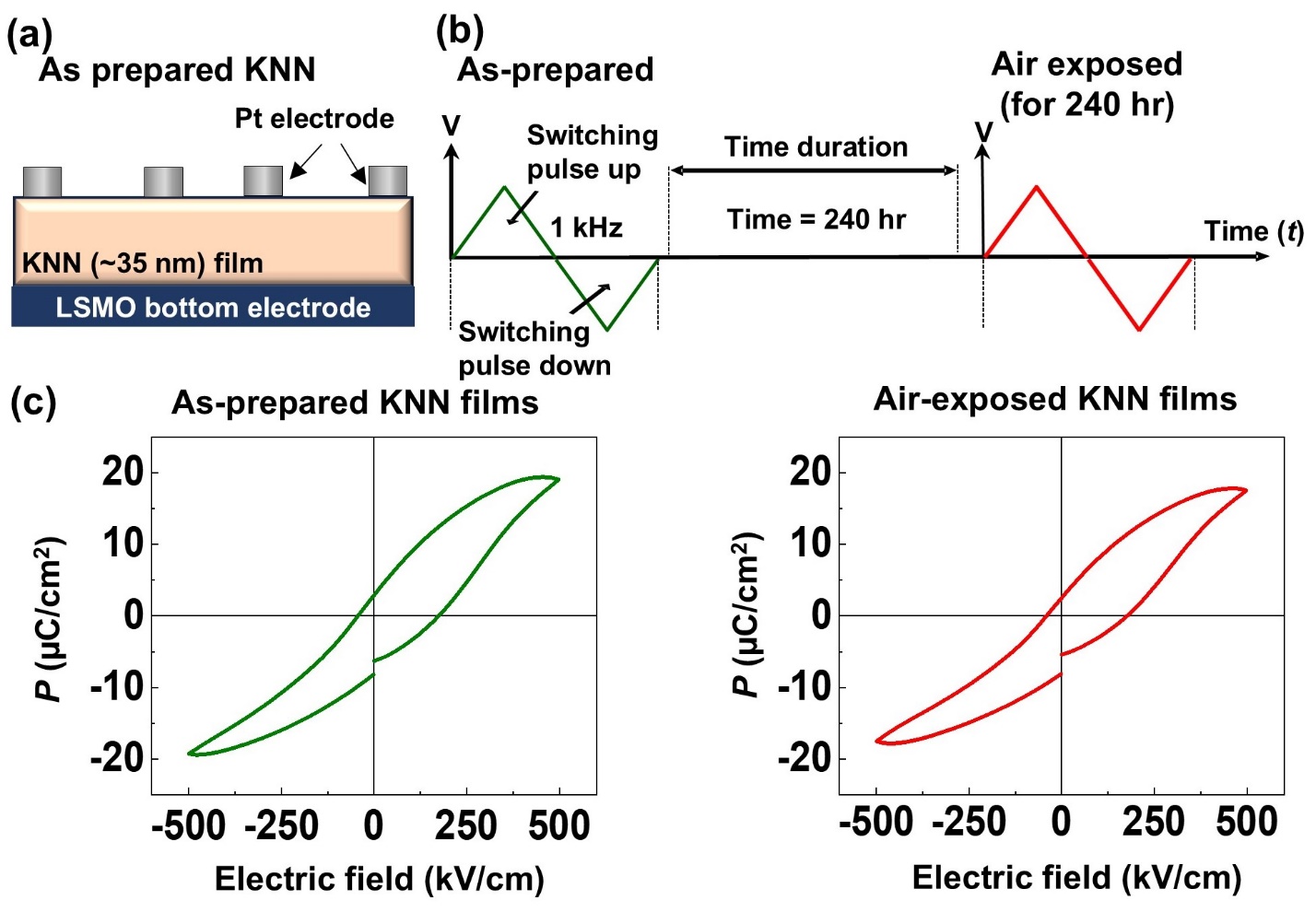


**Figure S13.** a) Schematic illustration of the polarization behavior in the as-grown and air-exposed states in the 2D planar geometry KNN (~35 nm) films. b) A schematic diagram of ferroelectric hysteresis loop measurement with a triangular pulse and a frequency of the 35 nm-thick KNN films. c) Corresponding *P*-*E* hysteresis loops of the films in the as-prepared and air-exposed case.

**Reversible control of ferroelectric hysteresis loops of KNN (~600 nm) films**


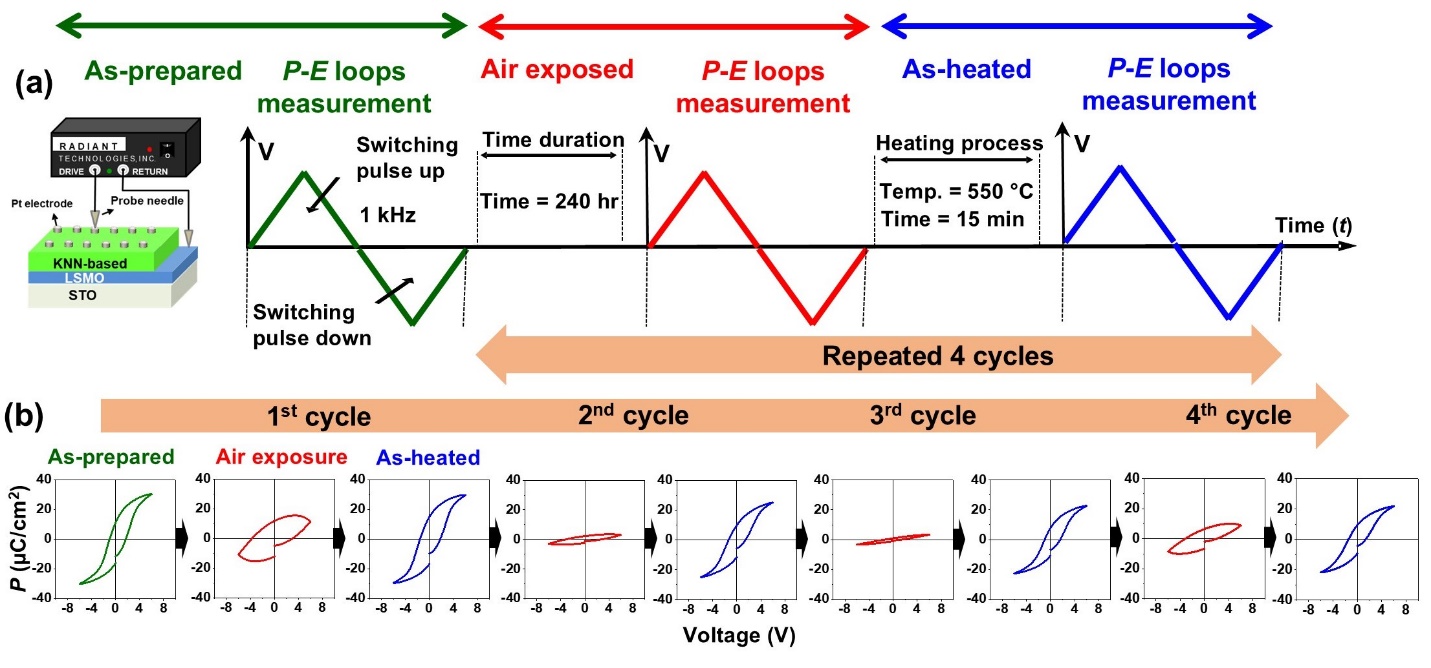


**Figure S14.** a) Schematic illustration of the measurement setup of the ferroelectric hysteresis loops in the as-prepared, air-exposed, and as-heated states. b) The ferroelectric polarization of the KNN (~600 nm) films in three states and reversible to the initial state upon thermal heating. The reversibility of the ferroelectric polarization response of the KNN (~600 nm) films was repeated 4 times between protonated and deprotonated states induced by air exposure and thermal annealing.

**Polarization retention loss in the presence of the domain wall depinning**

**Figure S15.** Schematic illustration of the polarization retention loss in the presence of the domain wall depinning. In the as-prepared, the domain walls (i.e., shown by a black dotted line) with the ferroelectric dipole directions (i.e., arrow in red) were shown in the enlarged view of the schematic for the nanopillar structure KNN films. The KNN films incorporating a certain amount of oxygen vacancies were represented with the dotted black circles in the Figure S15. In the presence of as-diffused hydrogen ions inside the nanopillar KNN films, tail-to-tail domain walls could be formed marked by blue arrows in the schematic figures, affecting the net polarization response and resulting impedance properties shown in the schematic Figure S15.

**Table S1.** The calculated lattice constants (i.e., in-plane and out-of-plane parameters) and the estimated lattice volume of 35 nm and 600 nm-thick KNN.

| Materials | Structure | Lattice parameters (Å) | | | Angle  *β* (°) | Estimated lattice volume (Å^3^) | References |
| --- | --- | --- | --- | --- | --- | --- | --- |
|  |  | In-plane (*a*) | | Out-of-plane (*c*) |  |  |  |
| Thinner KNN  (~35 nm) film | Pseudocubic | 3.905 | | 3.993 | N/A | 60.89 | This work |
| Thicker KNN  (~600 nm) film | Pseudocubic | 3.945 | | 4.001 | N/A | 62.27 | This work |
| K_0.5_Na_0.5_NbO_3_ single crystal | Orthorhombic (*Amm*2) | 3.976 | 3.932 | 3.969 | 90.000 | 62.06 | [5] |

**References**

[1] S. M. Yang, T. H. Kim, J.-G. Yoon, T. W. Noh, *Adv. Funct. Mater.* **2012**, *22*, 2310–2317.

[2] T. H. Kim, B. C. Jeon, T. Min, S. M. Yang, D. Lee, Y. S. Kim, S.‐H. Baek, W. Saenrang, C.‐B. Eom, T. K. Song, J.‐G. Yoon, T. W. Noh, *Adv. Funct. Mater.* **2012**, *22*, 4962–4968.

[3] H. Lee, T. H. Kim, J. J Patzner, H. Lu, J.-W. Lee, H. Zhou, W. Chang, M. K Mahanthappa, E. Y Tsymbal, A. Gruverman, C.-B. Eom, *Nano Lett.* **2016**, *16*, 2400−2406.

[4] D. Zhang, D. Sando, P. Sharma, X. Cheng, F. Ji, V. Govinden, M. Weyland, V. Nagarajan, J. Seidel, *Nat. Commun.* **2020**, *11*, 349.

[5] D. Lin, Z. Li, S. Zhang, Z. Xu, X. Yao, *J. Am. Ceram. Soc.* **2010**, *93*, 941–944.

[6] H. Liu, H. Wu, K. P. Ong, T. Yang, P. Yang, P. K. Das, X. Chi, Y. Zhang, C. Diao, W. K. A. Wong, E. P. Chew, Y. F. Chen, C. K. Ivan Tan, A. Rusydi, M. B. H. Breese, D. J. Singh, L.-Q. Chen, S. J. Pennycook, K. Yao, *Science* **2020**, *369*, 292–297.

[7] J. F. Scott, *J. Phys.: Condens. Matter*. **2008**, *20*, 021001.

[8] N. X. Duong, J.-S. Jang, M.-H. Jung, J.-S. Bae, C. W. Ahn, J. S. Jin, K. Ihm, G. Kim, S. Y. Lim, J. Lee, D. D. Dung, S. Lee, Y.-M. Kim, S. Lee, S. M. Yang, C. Sohn, I. W. Kim, H. Y. Jeong, S.-H. Baek, T. H. Kim, *Sci. Adv.* **2023**, *9*, eadd8328.
